# Correction: Cold exposure-induced plasma exosomes impair bone mass by inhibiting autophagy

**DOI:** 10.1186/s12951-024-02834-5

**Published:** 2024-09-18

**Authors:** Li-Min Lei, Fu-Xing-Zi Li, Xiao Lin, Feng Xu, Su-Kang Shan, Bei Guo, Ming-Hui Zheng, Ke-Xin Tang, Yi Wang, Qiu-Shuang Xu, Wen-Lu Ouyang, Jia-Yue Duan, Yun-Yun Wu, Ye-Chi Cao, Zhi-Ang Zhou, Si-Yang He, Yan-Lin Wu, Xi Chen, Zheng-Jun Lin, Yi Pan, Ling-Qing Yuan, Zhi-Hong Li

**Affiliations:** 1 National Clinical Research Center for Metabolic Disease, Hunan Provincial Key Laboratory of Metabolic Bone Diseases, Department of Metabolism and Endocrinology, The Second Xiangya Hospital, Central South University, Changsha, China; 2 Department of Radiology, The Second Xiangya Hospital, Central South University, Changsha, China; 3 Department of Cardiovascular Surgery, the Second Xiangya Hospital, Central South University, Changsha, China; 4 Department of Orthopaedics, The Second Xiangya Hospital, Central South University, Changsha, Hunan 410011 China; 5 Hunan Key Laboratory of Tumor Models and Individualized Medicine, The Second Xiangya Hospital, Central South University, Changsha, Hunan 410011 China; 6 Department of Endocrinology, The Second Affiliated Hospital of Kunming Medical University, No. 374 The Dianmian Avenue, Wuhua, Kunming, Yunnan 650101 China; 7 Department of Orthopaedics, Hunan Key Laboratory of Tumor Models and Individualized Medicine, The Second Xiangya Hospital, Central South University, Changsha, Hunan 410011 China

**Correction: Journal of Nanobiotechnology (2024) 22:361**

10.1186/s12951-024-02640-z

In this article, Zhi-Hong Li should have been denoted as a corresponding author. In this Correction article, Zhi-Hong Li has been denoted as corresponding author, in addition to Ling-Qing Yua. 

The original article has been corrected.

