# Correction: Cold exposure-induced plasma exosomes impair bone mass by inhibiting autophagy

**DOI:** 10.1186/s12951-025-03130-6

**Published:** 2025-02-28

**Authors:** Li-Min Lei, Fu-Xing-Zi Li, Xiao Lin, Feng Xu, Su-Kang Shan, Bei Guo, Ming-Hui Zheng, Ke-Xin Tang, Yi Wang, Qiu-Shuang Xu, Wen-Lu Ouyang, Jia-Yue Duan, Yun-Yun Wu, Ye-Chi Cao, Zhi-Ang Zhou, Si-Yang He, Yan-Lin Wu, Xi Chen, Zheng-Jun Lin, Yi Pan, Ling-Qing Yuan, Zhi-Hong Li

**Affiliations:** 1 National Clinical Research Center for Metabolic Disease, Hunan Provincial Key Laboratory of Metabolic Bone Diseases, Department of Metabolism and Endocrinology, The Second Xiangya Hospital, Central South University, Changsha, China; 2 Department of Radiology, The Second Xiangya Hospital, Central South University, Changsha, China; 3 Department of Cardiovascular Surgery, the Second Xiangya Hospital, Central South University, Changsha, China; 4 Department of Orthopaedics, The Second Xiangya Hospital, Central South University, Changsha, 410011 Hunan China; 5 Hunan Key Laboratory of Tumor Models and Individualized Medicine, The Second Xiangya Hospital, Central South University, Changsha, 410011 Hunan China; 6 Department of Endocrinology, The Second Affiliated Hospital of Kunming Medical University, No. 374 The Dianmian Avenue, Wuhua, Kunming, 650101 Yunnan China; 7 Department of Orthopaedics, Hunan Key Laboratory of Tumor Models and Individualized Medicine, The Second Xiangya Hospital, Central South University, Changsha, 410011 Hunan China

**Correction: Journal of Nanobiotechnology (2024) 22:361** 10.1186/s12951-024-02640-z

In this article, Fig. 8M (CT + antagomir NC group) appeared incorrectly as a duplicate of Figure 8M (CT + Vehicle group). For completeness and transparency, the old incorrect version and the corrected version of Figure 8 are displayed below. The original article has been corrected.

Incorrect Fig. 8

**Figure:**
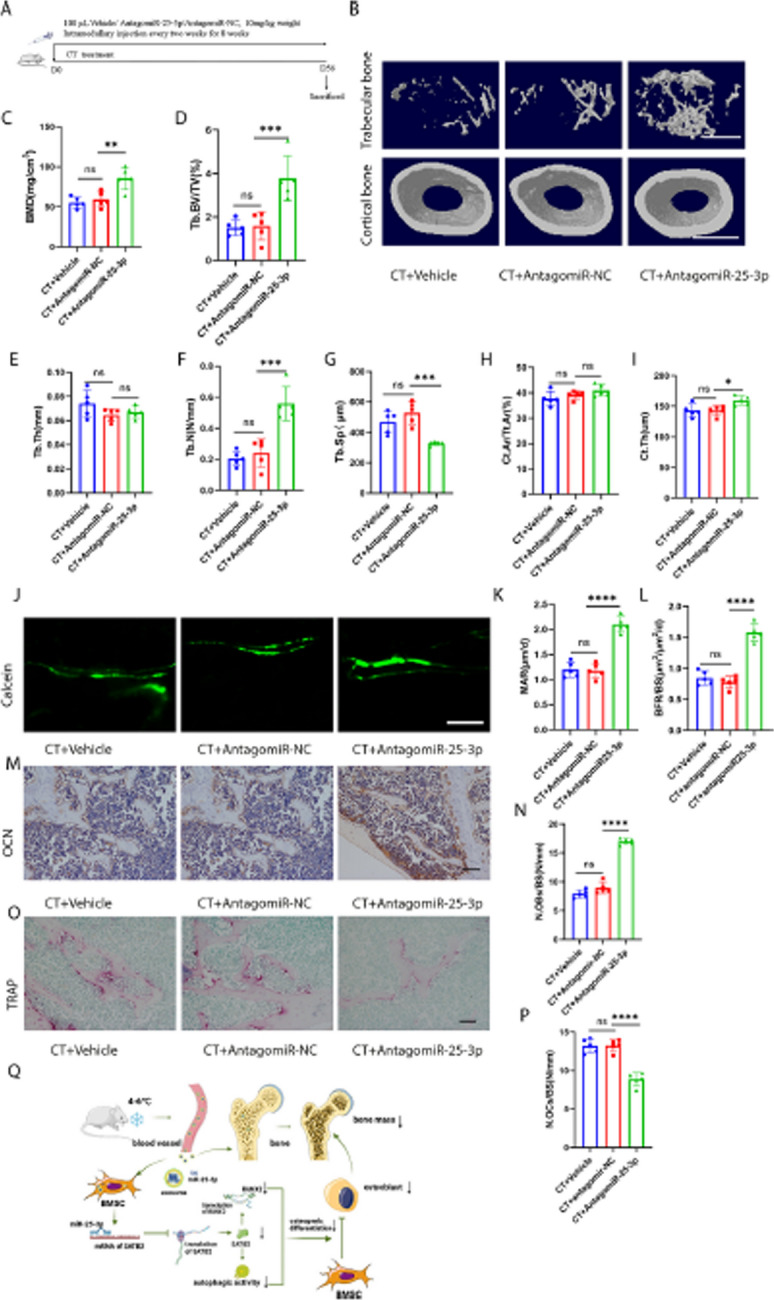
Inhibition of miR-25-3p level alleviated CT-induced bone loss. **A** Schematic flow diagram representing mice treated with CT + vehicle, CT + antagomiR-NC, and CT + antagomiR-25-3p. n = 5 per group. **B** Representative μCT images of trabecular (top) and cortical (bottom) bone in CT + vehicle, CT + antagomiR-NC-, and CT + antagomiR-25-3p-treated mice. Scale bars represent 500 μm (top) and 1 mm (bottom). **C**–**I** Parameters of trabecular bone mass analysed by micro-CT: BMD, Tb. BV/TV, Tb. Th, Tb. N, Tb. Sp, Ct. Ar/Tt. Ar, Ct. Th. n = 5 per group. **J** Calcein double labelling images of the mineralized surface of mouse femora. Scale bar represents 50 μm. **K**, **L** Parameters of bone formation MAR, BFR/BS. n = 5 per group. **M** Representative OCN-stained section. Scale bar represents 100 μm. **N** Quantification of the number of osteoblasts (N. OBs) on the trabecular bone surface (BS) in distal femora. n = 5 per group. **O** TRAP-stained sections. Scale bar represents 100 μm. **P** quantification of the number of osteoclasts (N. OCs) on the trabecular bone surface (BS) in distal femora. n = 5 per group. **Q** Mechanism of cold exposure induced bone loss. The vehicle referred to is PBS. * P < 0.05, ** P < 0.01, *** P < 0.001, **** P < 0.0001

Corrected Fig. 8

**Figure:**
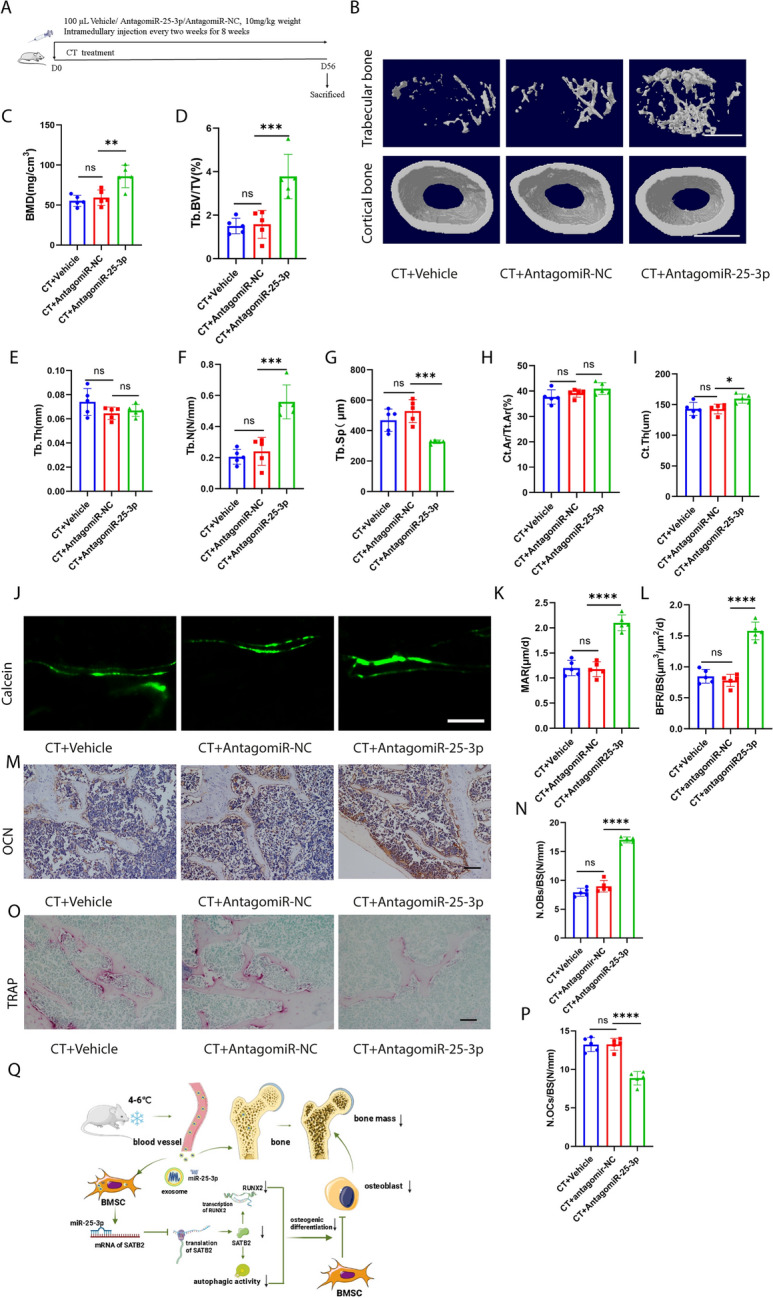
Inhibition of miR-25-3p level alleviated CT-induced bone loss. **A** Schematic flow diagram representing mice treated with CT + vehicle, CT + antagomiR-NC, and CT + antagomiR-25-3p. n = 5 per group. **B** Representative μCT images of trabecular (top) and cortical (bottom) bone in CT + vehicle, CT + antagomiR-NC-, and CT + antagomiR-25-3p-treated mice. Scale bars represent 500 μm (top) and 1 mm (bottom). **C**–**I** Parameters of trabecular bone mass analysed by micro-CT: BMD, Tb. BV/TV, Tb. Th, Tb. N, Tb. Sp, Ct. Ar/Tt. Ar, Ct. Th. n = 5 per group. **J** Calcein double labelling images of the mineralized surface of mouse femora. Scale bar represents 50 μm. **K**, **L** Parameters of bone formation MAR, BFR/BS. n = 5 per group. **M** Representative OCN-stained section. Scale bar represents 100 μm. **N** Quantification of the number of osteoblasts (N. OBs) on the trabecular bone surface (BS) in distal femora. n = 5 per group. **O** TRAP-stained sections. Scale bar represents 100 μm. **P** quantification of the number of osteoclasts (N. OCs) on the trabecular bone surface (BS) in distal femora. n = 5 per group. **Q** Mechanism of cold exposure induced bone loss. The vehicle referred to is PBS. * P < 0.05, ** P < 0.01, *** P < 0.001, **** P < 0.0001

