# Supplementary material for: Cold exposure-induced plasma exosomes impair bone mass by inhibiting autophagy
Source: J Nanobiotechnology. 2024 Jun 23;22:361. doi: 10.1186/s12951-024-02640-z (PMC11194967; doi:10.1186/s12951-024-02640-z)

**Cold exposure-induced plasma exosomes impair bone mass by inhibiting autophagy**

Li-Min Lei^1^, Fu-Xing-Zi Li^1^, Xiao Lin^2^, Feng Xu^1^, Su-Kang Shan^1^, Bei Guo^1^, Ming-Hui Zheng^1^, Ke-Xin Tang^1^, Yi Wang^1^, Qiu-Shuang Xu^1^, Wen-Lu Ouyang^1^, Jia-Yue Duan^1^, Yun-Yun Wu^1^, Ye-Chi Cao^1^, Zhi-Ang Zhou^3^, Si-Yang He^1^, Yan-Lin Wu^1^, Xi Chen^1^, [Zheng-Jun Lin](https://pubmed.ncbi.nlm.nih.gov/?sort=date&term=Lin+ZJ&cauthor_id=36864020)^4, 5^, Yi Pan^6^, Ling-Qing Yuan^1*^, Li Zhi-Hong^4, 5*^

1 National Clinical Research Center for Metabolic Disease, Hunan Provincial Key Laboratory of Metabolic Bone Diseases, Department of Endocrinology and Metabolism, The Second Xiangya Hospital, Central South University, Changsha, China,

2 Department of Radiology, The Second Xiangya Hospital, Central South University, Changsha, China

3 Department of Cardiovascular Surgery, the Second Xiangya Hospital, Central South University, Changsha, China.

1. Department of Orthopaedics, The Second Xiangya Hospital, Central South University, Changsha, Hunan, 410011, China.
2. Hunan Key Laboratory of Tumor Models and Individualized Medicine, The Second Xiangya Hospital, Central South University, Changsha, Hunan, 410011, China.
3. Department of Endocrinology, The Second Affiliated Hospital of Kunming Medical University, No. 374 The Dianmian Avenue, Wuhua, Kunming, Yunnan Province 650101, China.

***Corresponding authors** Ling-Qing Yuan, National Clinical Research Center for Metabolic Disease, Hunan Provincial Key Laboratory of Metabolic Bone Diseases, Department of Endocrinology and Metabolism, The Second Xiangya Hospital, Central South University, Changsha, China,

Eddress：allenylq@csu.edu.cn

telephone numbers: 13077371004

Zhi-Hong Li, Department of Orthopaedics, Hunan Key Laboratory of Tumor Models and Individualized Medicine, The Second Xiangya Hospital, Central South University, Changsha, Hunan, 410011, China.

Eddress: lizhihong@csu.edu.cn.

**Abstract**

Recently, environmental temperature has been shown to regulate bone homeostasis. However, the mechanisms by which cold exposure affects bone mass remain unclear. In our present study, we observed that exposure to cold temperature (CT) decreased bone mass and quality in mice. Furthermore, a transplant of exosomes derived from the plasma of mice exposed to cold temperature (CT-EXO) can also impair the osteogenic differentiation of BMSCs and decrease bone mass by inhibiting autophagic activity. Rapamycin, a potent inducer of autophagy, can reverse cold exposure or CT-EXO-induced bone loss. Microarray sequencing revealed that cold exposure increases the miR-25-3p level in CT-EXO. Mechanistic studies showed that miR-25-3p can inhibit the osteogenic differentiation and autophagic activity of BMSCs. It is shown that inhibition of exosomes release or downregulation of miR-25-3p level can suppress CT-induced bone loss. This study identifies that CT-EXO mediates CT-induced osteoporotic effects through miR-25-3p by inhibiting autophagy via targeting SATB2, presenting a novel mechanism underlying the effect of cold temperature on bone mass.

**Keywords: cold exposure, bone mass, exosomes, osteogenesis, autophagy, miR-25-3p**

**Introduction**

Osteoporosis is a common metabolic bone disorder marked by an imbalance between bone formation and resorption, leading to diminished bone mineral density and quality, rendering bones fragile and susceptible to fractures[[1](#_ENREF_1)]. Osteoporosis commonly arises from aging or estrogen deficiency, making elderly individuals, particularly postmenopausal women, the primary demographic affected[[2](#_ENREF_2)]. Other variables impacting bone loss, however, are still being investigated. The life history theory raises the idea that the prioritization of resources between biological items will depend on the environment[[3](#_ENREF_3)]. Environmental temperature plays a crucial role in physiological processes, necessitating continual adaptation to external temperature variations by organisms. Prior research has shown that environmental temperature significantly influences the regulation of immunity, lipid metabolism, and glucose metabolism[[4-8](#_ENREF_4)]. Recent evidence has also shown the associations between bone homeostasis and external temperature. Claire et al. found that warmth prevents bone loss through the gut microbiota[[9](#_ENREF_9)]. Exposure to warmth directly modulates cartilage growth, leading to increased limb length in homeotherms[[10](#_ENREF_10), [11](#_ENREF_11)]. A positive correlation has also been observed between fracture incidence and latitude[[9](#_ENREF_9)], which suggests that exposure to warmth has a beneficial effect on bone health. However, the potential impact of cold exposure on bone metabolism remains unexplored.

Exosomes (EXO) are extracellular vesicles measuring 40–150 nm, released by various cells and tissues[[12](#_ENREF_12), [13](#_ENREF_13)]. Initially, exosomes were characterized as 'garbage dumpsters' responsible for removing detrimental or unnecessary intracellular substances. Recently, exosomes have been recognized as 'signal boxes' responsible for facilitating communication between cells and organs[[14](#_ENREF_14)]. In recent years, exosomes have emerged as key players in bone metabolism, facilitating essential cell-to-cell communication mechanisms[[15-17](#_ENREF_15)].

As a dynamic recycling mechanism, autophagy generates fresh components and energy to facilitate cellular remodelling and maintain homeostasis[[18](#_ENREF_18)]. Autophagy is associated with numerous physiological and pathological processes, including development, differentiation, neurodegenerative diseases, stress, and infection[[19-22](#_ENREF_19)]. Recently, researchers have shown that autophagy regulates osteoblast differentiation, osteoclast differentiation, and chondrocyte differentiation[[23-26](#_ENREF_23)]. Additionally, decreased autophagic activity is observed during bone aging[[27](#_ENREF_27), [28](#_ENREF_28)], age-related osteopenia can be reversed through activation of autophagy[[29](#_ENREF_29), [30](#_ENREF_30)] and enhanced by autophagy suppression[[31](#_ENREF_31)]. As a result, autophagy plays a significant role in controlling bone metabolic homeostasis as a crucial physiological function. However, it remains unclear whether autophagy participates in the process of bone metabolism in cold temperature environments.

MicroRNAs (miRNAs) are short, noncoding RNAs that regulate various biological processes[[32](#_ENREF_32)], by interacting with complementary regions in the 3′ untranslated regions of target mRNAs and regulating the rate at which they promote their degradation[[33](#_ENREF_33)]. miRNAs can be encapsulated within exosomes and act in paracrine or endocrine manners on neighboring or distant cells[[34](#_ENREF_34)]. Exosomal miRNAs are also capable of regulating bone metabolism[[35](#_ENREF_35), [36](#_ENREF_36)]. Additionally, cold exposure can modify circulating miRNA levels[[37](#_ENREF_37)]. However, it remains unclear whether the altered circulating exosomal miRNA can impact bone metabolism.

In this study, we investigated whether cold exposure affects bone mass and the involvement of exosomes in regulating bone metabolism in cold-exposed mice. Furthermore, we discovered that autophagy plays a pivotal role in bone loss mediated by exosomes derived from the plasma of cold-exposed mice (CT-EXO). Mechanistically, we found that circulating exosomal miR-25-3p plays a significant role in cold exposure-induced bone loss by targeting SATB2 and inhibiting autophagy.

**Materials and methods**

**Cell isolation and culture**

As described previously, BMSCs were obtained from the bone marrow of the femurs and tibias of C57BL/6 mice[[38](#_ENREF_38)]. Initially, the femurs and tibias were separated under sterile conditions and placed in low-glucose DMEM supplemented with 1% penicillin/streptomycin (Gibco, Invitrogen, New York, USA). Subsequently, low-glucose DMEM containing 1% penicillin/streptomycin was utilized to flush out the bone marrow cavity. The cells were initially cultured in low-glucose DMEM supplemented with 15% fetal bovine serum (FBS) sourced from Bovogen, New Zealand, Australia, and 1% penicillin/streptomycin. Following a 72-hour incubation period, the culture medium was replaced with fresh complete low-glucose DMEM to eliminate non-adherent cells. Adherent cells were further cultured for 3–5 days and passaged until reaching 90% confluence. The harvested Bone Marrow Stromal Cells (BMSCs) were then seeded for experimental intervention. Upon reaching 80% confluence in the BMSC culture, the medium was substituted with osteogenic medium (Cat. No. MUCMX-90021; Cyagen BiosciencesInc, Guangzhou, China).

The acquisition of Bone Marrow Macrophages (BMMs) similarly originates from the bone marrow fluid. Initially, bone marrow was flushed out from the femurs and tibias of mice. After using the red blood cell lysis buffer, the harvested marrow cells underwent a 3-day culture in α-MEM complete medium (α-MEM + 10% FBS + 1% penicillin–streptomycin). Subsequently, the bone marrow cell suspension was collected, centrifuged, and resuspended in α-MEM complete medium supplemented with 30ng/ml m-CSF (Peprotech, Rocky Hill, USA) to obtain BMMs. These BMMs were then plated and cultured for an additional 3 days. Next, the BMMs were cultured in α-MEM complete medium containing 30ng/ml m-CSF and 50ng/ml RANKL (Peprotech, Rocky Hill, USA) for osteoclast induction. The medium was changed every other day. Osteoclast fusion was observed after 5-7 days, followed by TRAP staining. All cells were cultivated at 37 °C in a humidified 5% CO2 environment.

**Exosomes isolation and characterization**

After anesthetizing the mice, blood was drawn from the heart, collected in an anticoagulant tube, and centrifuged at 3000 g for 20 minutes to separate the plasma from the supernatant. Exosomes were isolated via ultracentrifugation. To eliminate cells and large debris, the plasma was then centrifuged at 10,000 g for 30 minutes at 4 °C. Subsequently, the supernatant was diluted threefold and centrifuged at 100,000 g for 70 minutes at 4 °C. The exosomes pellet was rinsed with 10 mL of 1× PBS before being centrifuged at 100,000 g for 70 minutes at 4 °C. The exosomes pellet was subsequently reconstituted in PBS and filtered through 0.22 μm filters (Millipore, USA) to obtain a sterile exosomes suspension. The concentration of exosomes was then determined using a BCA kit.

Transmission electron microscopy (TEM) from Hitachi, Tokyo, Japan, was utilized to assess the morphology and size of exosomes for characterization. Additionally, a molecular size analyzer (ZetaView PMX 110, Particle Metrix, Germany) was employed to measure the size distribution of the exosomes. The presence of exosomal markers, including TSG101, CD9, and CD81, was determined using western blot analysis.

**Exosomes uptake by BMSCs**

We labeled the exosomes with a green fluorescent dye (DiO, D5840, Solarbio) and co-incubated the labeled exosomes with BMSCs to confirm their uptake. In summary, 100 μg of exosomes was mixed with a solution of 6 μL of DiO dye (5 mM) in 1 mL of sterile PBS, followed by incubation at 37°C for 30 minutes. The mixture was then subjected to ultracentrifugation at 100,000 g for 70 minutes at 4°C to remove unbound dyes. Subsequently, the labeled exosomes were co-cultured with BMSCs for 12 hours at 37°C. After washing with PBS, the BMSCs were fixed in 4% paraformaldehyde for 30 minutes at room temperature. Subsequently, the BMSCs membrane was permeabilized with 0.5% Triton X-100 for 15 minutes and then washed three times. Phalloidin (red dye) (YP0052S, UElandy) was dissolved in 200 μL of PBS, and the BMSCs were stained with phalloidin for 40 minutes at room temperature. Following three rinses with PBS, the BMSCs were stained with DAPI (Beyotime Biotechnology, Shanghai, China) for 5 minutes at room temperature. Finally, the green, red, and blue fluorescent signals in the BMSCs were visualized using a confocal microscope (B43, Andor) after washing with PBS.

**Cell and exosomes transfection**

For cell transfection, miR-5-3p mimics, miR-25-3p inhibitor, SATB2 siRNA, and their corresponding control oligos (100 nM) were transfected into BMSCs using siRNA mate following the manufacturer’s instructions. miR-25-3p mimics, inhibitor, and their control oligos were obtained from Ribobio (Guangzhou, China), while SATB2 siRNA, control oligos, and siRNA mate (G04003) were purchased from GenePharma (Shanghai, China).

Ribobio Biotechnology (Guangzhou, China) provided the agomiRs and antagomiRs for exosome transfection. CT-EXOs were transfected with agomiR-25-3p, agomiR-NC, antagomiR-25-3p, or antagomiR-NC at a concentration of 200 nM for 30 minutes at 37°C. Excess agomiRs and antagomiRs were removed by centrifugation at 4,000 g for 5 minutes using a 100 kDa Amicon Ultra-4 Centrifugal Filter Unit (Millipore). The CT-EXOs transfected with agomiRs and antagomiRs were used in subsequent experiments.

**Western blot analysis**

Western blotting, as previously described, was used to determine protein expression[[39](#_ENREF_39)]. SDS-PAGE was used to load 30 μg of protein, which was then transferred to polyvinylidene fluoride membranes (Millipore, Billerica, MA, USA). The membrane was blocked with 5% non-fat milk for 1 hour before being incubated with primary antibodies, CD9 (ab92726, 1 : 1000, Abcam), CD81 (ab109201, 1 : 1000, Abcam), TSG101 (bs-1365R, 1 : 1000, Bioss), RUNX2 (ab23981, 1 : 2000, Abcam), GAPDH (10494-1-AP, 1 : 4000, Proteintech), COL-Ⅰ（14695-1-AP,1 : 1000, Proteintech），BMP2 (ab214821, 1 : 2000, Abcam), SATB2 (bs-1949R, 1 : 1000, Bioss), P62 (18420-1-AP, 1 : 2000, Proteintech), and LC3 (14600-1-AP, 1 : 2000, Proteintech), ATG5(66744-1-Ig,1 : 2000, Proteintech)，followed by incubation for 1 hour at room temperature with the secondary antibody conjugated to horseradish peroxidase. An Amersham Imager 600 analyser (General Electric, USA) and an enhanced chemiluminescence reagent (Millipore, Billerica, MA) were used to image the immunoreactive bands.

**Alizarin Red staining**

BMSCs were seeded in 24-well plates and treated with intervention factors for 14 days. After washing with PBS, the BMSCs were fixed with 4% paraformaldehyde for 30 minutes at room temperature. Subsequently, the cells were washed three times with PBS and stained with Alizarin Red staining solution (Servicebio, Wuhan, China) for 5 minutes at room temperature. Finally, the cells were observed and photographed under a microscope after further washing with PBS.

**SA-β-gal assay**

Senescence-associated β-galactosidase (SA-β-gal) staining was done according to the manufacturer’s instructions using an SA-β-gal staining kit (C0602, Beyotime). In brief, digital images of randomly selected fields were captured and analyzed using an image analysis tool (BioQuant). The percentage of SA-β-gal-positive cells was calculated for each sample to determine the proportion of senescent cells.

**Colony formation experiment**

BMSCs were suspended and planted into 6-well plates at a density of 200 cells. The cells were cultured for 10 days in a complete medium. Cell colonies were washed three times with PBS, fixed with 4% PFA for 30 minutes at room temperature, then stained with 0.5% crystal violet solution for 30 minutes at room temperature. The excess stain was then wiped away, and the plates were air-dried.

**RNA sequencing**

RNA sequencing was performed on the RT-EXO and CT-EXO groups (with a sample size of three for each group). A NanoDrop spectrophotometer and Agilent 2100 bioanalyser (Santa Clara, CA, USA) were used to extract and quantify total RNA. On a BGIseq500 platform (BGI, Shenzhen, China), a Phi29-amplified mRNA library produced 100 base pair reads. SOAPnuke (V1.5.2) was used to filter the sequencing data, and Bowtie2 (V2.2.5) was used to compare the clean reads with the gene database created by the Shenzhen Beijing Genomics Institute. This was done to calculate gene expression levels and find differentially expressed genes (DEGs) (fold change > 1.5, P < 0.05).

**Quantitative real-time polymerase chain reaction (qRT-PCR) analysis**

RNA was isolated from exosomes according to the manufacturer’s instructions using a miRNeasy® Mini kit (Qiagen, Cat. No. 217084). TRIzol reagent (Invitrogen, Carlsbad, USA) was used to extract cellular total RNA. Reverse transcription and qRT-PCR amplification of RNA isolated from exosomes and cells were carried out in accordance with the manufacturer’s instructions using a Mir-X miRNA First-Strand Synthesis Kit (Takara, Japan, Cat. No. 638315). From GeneCopoeia, the following 5' miRNA-specific primers were ordered. The 2^−ΔΔCT^ method was used for relative quantification, and U6 small nuclear RNA was used as the standard for normalization.

**Plasmid constructs and luciferase reporter assay**

As well as predicted miR-25-3p binding sites, wild-type and mutant portions of the SATB2 3′-UTR were cloned into the PmeI and XbaI restriction sites of the pGL3 luciferase reporter vector (Promega). Stratagene’s QuikChange Site-Directed Mutagenesis Kit was used to create a mutant form of the SATB2 3′-UTR. The miR-25-3p mimics or control and a luciferase reporter carrying the wild-type or mutant SATB2 3′-UTR were co-transfected into BMSCs. Using luciferase assay equipment (Promega, USA), the luciferase activity was discovered 48 hours after transfection. The relative luciferase activity of cells was normalized using the *Renilla* luciferase activity.

**Animals**

C57BL/6 female mice aged 12 weeks were used in this investigation, and they were randomized to several treatment groups. Cold-exposed mice were placed in a 4–6 °C environment for 8 weeks to evaluate how cold exposure impacts bone mass, while the room-exposure mice were raised at room temperature of around 22 °C. To test the role of exosomes in CT-induced bone loss, mice were given 2 mg/kg of GW4869 (S7609, Selleck) or vehicle every other day for 8 weeks by intraperitoneal injection, and the vehicle represented DMSO. We treated the mice with cold exposure-induced plasma or exosomes removed from cold exposure-induced plasma (100 μL) twice a week for 8 weeks in RT mice through tail vein injection, to test the effects of CT-plasma or CT-EXO on bone. Moreover, the plasma was centrifuged at 100,000 *g* for 18 hours at 4 °C to remove the exosomes. The mice that were treated with PBS served as the control group. To determine whether cold exposure-induced plasma-derived exosomes influence bone mass, RT mice were given 200 μg CT-EXO or RT-EXO through tail vein injection. To detect whether autophagy influences cold exposure-induced plasma-derived exosomes-induced bone loss, rapamycin- or vehicle-treated animals were given CT-EXO or an equal volume of PBS. We also intervened with RT-EXO in CT mice to observe its effects on the mice. To investigate whether autophagy is involved in cold exposure-induced bone loss, CT mice were given rapamycin (S1039, Selleck) or Vehice every other day for 8 weeks by intraperitoneal injection, and the vehicle represented DMSO. We also observed the effects of RAP on CT-EXO intervention in RT mice.To determine the role of miR-25-3p in CT-induced bone loss, we administered antagomiR-25-3p or antagomiR-NC at 10 mg/kg or vehicle once every 2 weeks by tail vein injection or Intramedullary injection, for 8 weeks,.

**Tracing of exosomes *in vivo***

The plasma-derived exosomes were labeled with DiR dye (Invitrogen, USA) and administered to the mice via tail vein injection following the manufacturer’s instructions. Specifically, DiR was dissolved in ethanol to prepare a working solution of 200 μg/mL. Five microliters of DiR working solution was added to 100 μg of exosomes and incubated for 1 hour at room temperature in the dark. The unbound dyes and ethanol were removed by ultracentrifugation at 110,000 g for 70 minutes at 4°C, and the pellet was resuspended in PBS to achieve a concentration of 100 μg exosomes per 100 μL PBS. Mice were injected with DiR-labeled exosomes, PBS, or DiR solution (100 μL per mouse) via the tail vein, and imaging was performed on the mice, as well as the femur and tibia, 12 hours after injection.

To track exosomes in bone tissues, we labeled the exosomes with a green fluorescent dye (DiO, D5840, Solarbio) before injecting them into mice. Femur samples were collected for detection after 24 hours. Following a 1-day fixation in 4% paraformaldehyde (PFA), the femur underwent freeze-sectioning after decalcification in 0.5 M EDTA for 4 days. The sections were then washed three times in PBS before staining the nuclei with DAPI (Beyotime Biotechnology, Shanghai, China) for 5 minutes at room temperature. Subsequently, the green and blue fluorescent signals in the sections were visualized using a confocal microscope (B43, Andor) following PBS washing.

**μCT analysis**

The femur samples were fixed in 4% PFA for 2 days prior to scanning using a vivaCT 80 μCT scanner (voltage: 70 kV; current: 400 μA; X-ray tube potential: 55 kVp; integration time: 400 ms; voxel size: 11.4 μm; SCANCO Medical AG, Bruttisellen, Switzerland). The characteristics of the distal femoral metaphyseal trabecular bone and the diaphyseal cortical bone were assessed using image reconstruction software (NRecon), data analysis software (CTAn v1.11), and 3D model visualization software (CTVol v2.2). The trabecular bone analysis focused on a region of interest (ROI) covering 5% of the length of the femur, beginning 0.3 mm proximal to the distal growth plate. Parameters including bone mineral density (BMD), trabecular bone volume fraction (Tb. BV/TV), trabecular number (Tb. N), trabecular thickness (Tb. Th), and trabecular separation (Tb. Sp) were calculated. For cortical bone analysis, the ROI was extended proximally for 10% of the femoral length, starting at 40% of the femoral length proximal to the distal growth plate. Quantitative measurements included cortical bone area fraction (Ct. Ar/Tt. Ar), cortical area (Ct. Ar), total area (Tt. Ar), and cortical thickness (Ct. Th).

**Tissue staining assays**

The femurs were fixed with 4% PFA for 2 days, and then decalcified in 0.5 M EDTA for 2 weeks before being embedded in paraffin. As previously disclosed[[40](#_ENREF_40)], longitudinal slices of bone samples, 5 µm thick, were prepared and stained for osteocalcin (OCN) and tartrate-resistant acid phosphatase (TRAP). Immunohistochemical staining for OCN was performed using a Universal Two-Step Immunohistochemistry Kit (zsbio, Beijing, China) following the manufacturer’s instructions. Proteintech supplied the anti-OCN antibody (23418-1-AP), and Solarbio provided the TRAP staining kit (G1492). The number of OCN- or TRAP-positive cells was quantified using Image-Pro Plus 6.

**Dynamic bone formation**

To evaluate dynamic bone formation, mice were administered 0.1% calcein (Sigma-Aldrich; 10 mg/kg body weight) in PBS 9 and 3 days prior to sacrifice. Tibias were then harvested, fixed in 4% PFA for 2 days, dehydrated in 70% ethanol, and embedded in methyl methacrylate. Subsequently, calcein double labeling was observed under a fluorescence microscope after longitudinal sectioning of undecalcified bones into 10 μm-thick slices. Bone formation rate (BFR)/bone surface (BS) and mineral apposition rate (MAR) were analyzed throughout the entire region of interest (ROI) using Image-Pro Plus 6 software.

**Statistical analysis**

All data are presented as mean ± SD. Statistical analysis was performed using GraphPad Prism version 8.0. Student’s t-test was utilized to compare results between two groups, while one-way ANOVA followed by Dunnett’s test was employed for comparisons involving more than two groups. A significance level of P < 0.05 was considered statistically significant. Representative experimental results are depicted in the figures, with each experiment being conducted at least three times.

**Results**

**Cold exposure induces bone loss and** **alters bone metabolism in mice**

To directly investigate the impact of cold exposure on bone metabolism, C57BL/6 mice were subjected to cold temperature (CT; 4–6 °C) for 8 weeks, starting at 12 weeks of age, while control groups were maintained at room temperature (RT; 22–24 °C) throughout the experiment (Fig. 1A). Subsequently, mice were euthanized, and their bone tissues were prepared for further analysis. Micro-computed tomography (μCT) analysis of femurs revealed that CT mice exhibited significantly reduced bone mass and more compromised bone microstructures compared to RT mice. Specifically, CT mice showed significantly lower bone mineral density (BMD), trabecular bone volume fraction (Tb. BV/TV), trabecular number (Tb. N), and trabecular thickness (Tb. Th), as well as a trend of increased trabecular separation (Tb. Sp), without altering cortical bone area fraction (Ct. Ar/Tt. Ar), but with a decrease in cortical thickness (Ct. Th) (Fig. 1B–I). Calcein double labeling demonstrated impaired new bone formation and mineralization in CT mice, as indicated by bone formation rate per bone surface (BFR/BS) and mineral apposition rate (MAR) values (Fig. 1J–L). OCN immunohistochemical staining revealed fewer osteoblasts on the trabecular bone surface in CT mice compared to RT mice (Fig. 1M, N). TRAP staining showed a significant increase in the number of osteoclasts due to cold exposure (Fig. 1O, P). Additionally, cold exposure led to reductions in femur length and body weight (Fig. S1A–C). These findings suggest that cold exposure disrupts bone metabolism by inhibiting osteoblastic bone formation and increasing osteoclastic bone resorption, resulting in decreased bone mass.

**Inhibition of exosomes release alleviated CT-induced bone loss**

To further investigate the involvement of exosomes in CT-induced bone loss, we systematically inhibited exosomes release in mice exposed to cold temperatures. We utilized the pharmaceutical drug, GW4869, known for its efficient suppression of exosome biogenesis/release, to treat CT mice (Fig. 2A). Plasma was collected from mice, exosomes were extracted, and nanoparticle tracking analysis (NTA) was conducted. We confirmed that GW4869 significantly reduced the number of plasma exosomes (Fig. S2A). Bone marrow macrophages (BMMs) were isolated from GW4869- or vehicle-treated CT mice, and it was observed that GW4869 did not alter the osteoclastogenic activity of BMMs from CT mice (Fig. S2B, C). However, we found an increase in osteogenic activity in bone marrow mesenchymal stem cells (BMSCs) from GW4869-treated CT mice compared to those from CT mice, as evidenced by Alizarin Red S (ARS) staining and the expression of COL-1, RUNX2, and BMP2 (Fig. S2D-G). As expected, administration of GW4869 in CT mice effectively improved bone microarchitecture, resulting in increased BMD, Tb. BV/TV, Tb. N, Tb. Th, Ct. Ar/Tt. Ar, and Ct. Th, as well as decreased Tb. Sp, compared to vehicle-treated CT-induced mice (Fig. 2B–I). GW4869 also increased new bone formation and mineralization in CT mice, as shown in Fig. 2J–L. While GW4869 enhanced the number of osteoblasts on the trabecular bone surface of CT mice, indicated by OCN immunohistochemical staining, it had no effect on the number of osteoclasts, as indicated by TRAP staining (Fig. 2M–P). Additionally, GW4869 increased the length of the femur in CT mice (Fig. S3A, B) and elevated the body weight of CT mice (Fig. S3C). These experimental findings suggest that inhibiting the release of exosomes can significantly reduce the loss of bone mass caused by cold exposure, indicating a potential significant role of exosomes in CT-induced bone loss.

**Plasma from CT-exposed mice can reduce bone mass, but removing exosomes alleviates CT-plasma-induced bone loss**

The above experiments demonstrated the essential role of exosomes in cold exposure-induced bone loss. Thus, we aimed to investigate the effects of plasma and exosomes in plasma on bone metabolism during cold exposure. We treated the RT mice with plasma from CT mice (CT-plasma) or plasma from CT mice from which exosomes had been removed (CT-plasma^-EXO free^) by ultracentrifugation (Fig. 3A). As shown in Fig. 3B–I, CT-plasma impaired BMD, Tb. BV/TV, Tb. N, Tb. Th, and Ct. Th, as well as increasing Tb. Sp, compared to the control group mice. Calcein double labelling revealed the impairment of new bone formation and mineralization in the CT-plasma-treated mice (Fig. 3J–L). When comparing CT-plasma-treated mice to control mice, OCN immunohistochemistry staining revealed a reduced number of osteoblasts on the trabecular bone surface (Fig. 3M, N). TRAP staining showed that CT-plasma increased the number of osteoclasts (Fig. 3O, P). However, when the RT mice were treated with CT-plasma^-EXO free^, the bone mass and bone microstructures were improved compared with CT-plasma-treated mice (Fig. 3B–I). Furthermore, removing the exosomes also ameliorated new bone formation and mineralization (Fig. 3J–L). After removing the exosomes, the impact of CT-plasma on osteoblasts and osteoclasts was also ameliorated (Fig. 3M–P). Furthermore, we observed that CT-plasma reduced femur length; however, this effect was attenuated upon exosomes removal (Fig. S4A, B). However, no significant difference in body weight was observed among these groups (Fig. S4C). These data indicate that CT-plasma plays a pivotal role in CT-induced bone loss, with CT-EXO exerting significant effect.

**Plasma-derived exosomes from CT-exposed mice can be taken up by BMSCs and** **impair their osteogenic differentiation**

To further elucidate the role of plasma exosomes in cold-induced bone loss, we isolated exosomes from the plasma of CT or RT mice to investigate their involvement in the osteogenic development of BMSCs. Transmission electron microscopy (TEM) analysis confirmed the morphology of exosomes isolated from plasma, showing a cup- or sphere-shaped structure (Fig. 4A). NTA revealed that the size of these particles ranged mostly from 40 nm to 150 nm (Fig. 4B), consistent with previously reported exosome size distributions [[41](#_ENREF_41)]. Additionally, exosomal marker proteins CD9, CD81, and TSG101 were found to be highly abundant in plasma-derived exosomes (Fig. 4C).

To investigate the potential influence of plasma-derived exosomes on BMSCs function, we assessed their uptake by BMSCs. Plasma-derived exosomes were labeled with DiO and then co-incubated with BMSCs. Both exosomes from the plasma of room temperature-exposed mice (RT-EXO) and cold temperature-exposed mice (CT-EXO) were found to be taken up by BMSCs, as demonstrated in Fig. 4D. BMSCs were treated with RT-EXO and CT-EXO at a dose of 100 μg/mL. Interestingly, CT-EXO significantly suppressed osteogenic differentiation, as evidenced by a decrease in RUNX2 protein levels (Fig. 4E) and Alizarin Red staining (Fig. 4F–H). Conversely, RT-EXO stimulated osteogenic differentiation of BMSCs (Fig. 4E–H). Furthermore, as indicated in Fig. 4I, RT-EXO treatment promoted colony formation, while CT-EXO decreased colony formation. Additionally, CT-EXO may increase BMSCs senescence, as evidenced by more SA-β-gal-positive BMSCs, whereas RT-EXO inhibited BMSCs senescence (Fig. 4J). These findings suggest that CT-EXO play a significant role in BMSCs osteogenic differentiation. Moreover, we observed that CT-EXO have a promoting effect on osteoclast differentiation of BMMs, as demonstrated by TRAP staining (Fig. S5A, B). These data indicate that CT-EXO significantly inhibit the osteogenic differentiation ability of BMSCs and have a certain promoting effect on osteoclast differentiation of BMMs. However, since the earlier finding that inhibition of exosomes release did not significantly inhibit osteoclast differentiation in vivo, we primarily focused our research on the effect of CT-EXO on osteogenic differentiation of BMSCs.

**CT-EXO reduced bone mass and disrupted bone metabolism in RT mice, while RT-EXO increased bone mass in RT mice and mitigated CT-induced bone loss**

DiR-labelled plasma-derived exosomes were injected into mice via the tail vein, and their biodistribution was monitored in vivo to assess uptake by bone tissues. Fluorescence was primarily observed in the liver and spleen (Fig. 5A and S6A). We hypothesize that the intense fluorescence signals emitted by the liver and spleen masked the signals originating from bone tissues. Consequently, we performed distinct fluorescence imaging of bone tissues. Subsequently, upon sacrificing the mice, we isolated their tibias and femora to examine the fluorescent signals. Both the tibia and femur displayed robust fluorescent signals (Fig. 5B), indicating successful injection of DiR-labelled exosomes via the tail vein and their delivery to bone tissue. To examine exosomes distribution at the microscopic level, a green fluorescent dye (DiO) was employed to label exosomes. Fluorescent signals in tissues from mice receiving labelled exosomes via the tail vein were observed using a fluorescence microscope, revealing the presence of DiO-labelled exosomes in the liver and spleen (Fig. S6B, C). The presence of DiO-labelled exosomes in bone tissues is illustrated in Fig. 5C, suggesting that plasma-derived exosomes might target bone cells and modulate bone metabolism.

Then, we investigated whether direct administration of PBS, RT-EXO and CT-EXO could influence bone mass and alter bone metabolism in mice cultured in room temperature (RT). To determine the optimal dosage of exosomes for intervention, we pre-treated RT mice with various doses of CT-EXO (Fig. S7A). We observed that the most significant reduction in bone mass occurred with an intervention of 200 μg of CT-EXO (Fig. S7B-I). Consequently, we selected this dosage for subsequent experiments. Subsequently, the RT mice received treatments with RT-EXO or CT-EXO twice a week for 8 weeks (Fig. 5D). Micro-computed tomography (μCT) analysis revealed that administration of RT-EXO potentially improved bone density and enhanced bone microarchitecture (Fig. 5E–J). Conversely, CT-EXO significantly induced bone loss and disrupted bone microarchitecture, as evidenced by significantly lower BMD, Tb. BV/TV, Tb. N, Tb. Th, Ct. Th, and higher Tb. Sp compared to mice treated with RT-EXO (Fig. 5E–J). There was no statistically significant difference in cortical bone between the groups (Fig. 5K, L). Calcein double labeling showed that RT-EXO administration might stimulate new bone formation and mineralization in mice compared with those treated with PBS, whereas CT-EXO treatment led to a significant decrease in new bone formation and mineralization compared to PBS- or RT-EXO-treated mice (Fig. 5M–O). OCN immunohistochemical staining revealed a greater number of osteoblasts on the trabecular bone surface of RT-EXO-treated mice compared to the PBS group, whereas CT-EXO significantly decreased the number of osteoclasts in mice compared with PBS- or RT-EXO-treated mice (Fig. 5P, Q). CT-EXO induced significant increases in the number of osteoclasts, as determined by TRAP staining (Fig. 5R, S). However, a decrease of the number of osteoclasts was not observed in mice administered with RT-EXO (Fig. 5R, S). We also found that CT-EXO decreased the length of the femur, but RT-EXO increased it (Fig. S8A, B). However, there was no significant difference in body weight in these groups (Fig. S8C). The preceding results indicate that CT-EXO can substantially reduce the bone mass of RT mice.

To investigate the potential of RT-EXO in mitigating CT-induced bone loss, CT mice were administered either PBS or RT-EXO via tail vein injection twice weekly for 8 weeks (Fig. S9A). μCT analysis revealed that RT-EXO administration effectively attenuated bone loss induced by cold exposure and enhanced bone microarchitecture, as evidenced by the increased BMD, Tb. BV/TV, Tb. N, and Tb. Th, and the decreased Tb. Sp in comparison to the PBS-treated CT mice (Fig. S9B-G). There was an increase in Ct. Ar/Tt. Ar, but not Ct. Th (Fig. S9H, I). Calcein double labelling revealed an increase in new bone formation and mineralization in CT mice following RT-EXO treatment (Fig. S9J–L). Immunohistochemistry staining for osteocalcin (OCN) indicated an increase in the number of osteoblasts on the trabecular bone surface in CT mice treated with RT-EXO (Fig. S9M, N). However, as shown by the TRAP staining, treatment with RT-EXO did not reduce the number of osteoclasts on the trabecular bone surface (Fig. S9O, P). Additionally, RT-EXO administration was found to increase femur length (Fig. S9Q and R), while no significant difference in body weight was observed between the experimental groups (Fig. S9S). These results collectively suggest a significant role for plasma-derived exosomes in regulating bone metabolism in mice subjected to both RT and CT environments. Furthermore, it is plausible that CT-EXO may mediate bone loss induced by cold exposure.

**CT-EXO diminishes bone mass through the inhibition of autophagy, whereas rapamycin demonstrates the potential to reverse cold exposure-induced bone loss**

Given that age-related bone loss correlates with diminished autophagic activity, we investigated the impact of CT-EXO on the senescence of bone marrow-derived mesenchymal stem cells (BMSCs). Subsequently, we evaluated the expression of P21 in the bone tissues of mice subjected to either room temperature (RT) or cold exposure. Cold exposure markedly hastened the senescence of bone tissues, as evidenced by a notable elevation in P21 expression, as depicted in Fig. 6A. We further observed that cold exposure suppresses autophagic activity, as evidenced by elevated expression of P62 and a decreased LC3II:LC3I ratio in bone tissues (Fig. 6A). Intriguingly, our investigation revealed that CT-EXO significantly attenuated the autophagic activity of BMSCs, as indicated by increased P62 expression, a reduced LC3 II:LC3I ratio, decreased levels of ATG5, and diminished formation of autophagosomes (Fig. S10A-B). Conversely, RT-EXO did not exert a significant impact on the autophagic activity of BMSCs (Fig. S10A-B). We determined that CT-EXO could inhibit the autophagic activity of BMSCs and it can be reversed by rapamycin (RAP), a kind of autophagy agonist, by detecting the autophagy markers, P62 and LC3 (Fig. 6B), and detecting the autophagosome formation by transmission electron microscopy (Fig. 6F). Furthermore, as depicted in Fig. 6B, RAP also effectively reversed the CT-EXO-induced inhibition of RUNX2 expression in BMSCs. Alizarin Red staining further illustrated that RAP enhances the osteogenic differentiation of BMSCs and mitigates the impairment of BMSCs' osteogenic differentiation induced by CT-EXO (Fig. 6C–E). Furthermore, RAP also mitigated the CT-EXO-induced senescence of BMSCs, as evidenced by the expression of P21 and SA-β-gal staining (Fig. 6B, G). As indicated by the findings, CT-EXO inhibits osteogenic differentiation and induces senescence of BMSCs by suppressing autophagy.

We subsequently investigated whether enhancing autophagic activity could enhance the bone mass of CT mice. We administered varying doses of RAP to CT mice (Fig. S11A) and observed that a dosage of 2 mg/kg body weight significantly augmented the bone mass of CT mice (Fig. S11B-I). Therefore, we opted for this dosage for subsequent experiments. CT mice were administered RAP or vehicle (Fig. S12A). As depicted in Fig. S12B, RAP augmented the autophagic activity of bone tissues, evidenced by reduced expression of P62, elevated expression of ATG5, and an increased LC3 II:LC3 I ratio. Subsequent μCT analysis revealed that RAP treatment elevated BMD, Tb. BV/TV, Tb. Th, and Tb. N in CT mice, while reducing Tb. Sp (Fig. S12C–H). Figures S12I and J demonstrate that RAP had no effect on Ct. Ar/Tt. Ar but increased Ct. Th in CT mice. Additionally, as depicted in Fig. S12K–M, RAP exhibited a protective effect on new bone formation and mineralization in CT mice. RAP treatment resulted in an increase in the number of osteoblasts in CT mice (Fig. S12N, O), while TRAP staining revealed no significant differences in osteoclasts between the two groups (Fig. S12P, Q). Moreover, RAP increased the length of the femur in CT mice (Fig. S12R, S) and did not affect the body weight of CT mice (Fig. S12T).

To assess whether autophagy contributes to CT-EXO-induced bone loss, we administered vehicle, CT-EXO, RAP, or CT-EXO+RAP to mice (Fig. 6H). The µCT results indicated that RAP significantly improved bone mass and bone microarchitecture (Fig. 6I-N), which is consistent with the results of Ma et al.[[29](#_ENREF_29)]. Furthermore, we observed that RAP exerted a protective effect on bone microarchitecture and bone mass in mice treated with CT-EXO (Fig. 6I–N). No statistically significant difference in cortical bone was noted between groups (Fig. 6O, P). Calcein double labeling revealed that RAP enhanced new bone formation and mineralization, effectively reversing the CT-EXO-induced impairment of new bone formation and mineralization (Fig. 6Q–S). OCN staining demonstrated that RAP increased the number of osteoblasts in CT-EXO-treated mice (Fig. 6T, U). However, TRAP staining revealed that RAP had no significant effect on osteoclast numbers, regardless of CT-EXO treatment (Fig. 6V, W). Additionally, we found that CT-EXO decreased the autophagic activity of bone tissues, as indicated by higher expression of P62, lower expression of ATG5, and a decreased LC3II:LC3I ratio, and this effect could be reversed by RAP (Fig. S13A). Subsequently, BMSCs were isolated from the different groups of mice. Our results indicated that CT-EXO significantly impaired the osteogenic differentiation of BMSCs, while RAP was able to restore their osteogenic differentiation ability, as evidenced by the expression of COL-1, RUNX2, and BMP2 (Fig. S13B). Furthermore, Fig. S13C and D demonstrate that RAP could also reverse the reduction in femur length induced by CT. However, there was no significant effect on body weight in these groups (Fig. S13E).

These results suggest that decreased autophagic activity contributes to bone loss induced by CT-EXO or CT. Furthermore, the autophagy of BMSCs appears to play a role in the impact of CT-EXO on osteogenic differentiation.

**Exosomal miR-25-3p enriched in CT-EXO regulated osteogenic differentiation and autophagy by tageting SATB2 in BMSCs**

To delve deeper into the mechanism underlying the detrimental effects of CT-EXO on bone loss, we employed microarray-based miRNA expression profiling analysis to identify differential abundance of miRNAs between RT-EXO and CT-EXO. Our analysis revealed that CT-EXO exhibited 33 significantly upregulated miRNAs and 38 significantly downregulated miRNAs (fold change ≥ 2 and P ≤ 0.05) (Fig. 7A). qRT-PCR was subsequently employed to validate the differential abundance of three miRNAs related to osteogenesis and autophagy (miR-205-5p, miR-30d-5p, miR-320-3p, miR-222-3p, and miR-25-3p) in plasma exosomes. Among these, miR-25-3p was found to be the most abundant in CT-EXO compared to RT-EXO (Fig. 7B–F). Consequently, we selected miR-25-3p, which was highly abundant in CT-EXO, for further investigation.

To investigate the role of miR-25-3p in BMSCs osteogenic differentiation and autophagy, BMSCs were treated with CT-EXO transfected with either agomiR-25-3p or antagomiR-25-3p. agomiR-NC and antagomiR-NC, respectively serving as negative controls for agomiR-25-3p or antagomiR-25-3p, were also transfected into CT-EXO. As depicted in Fig. 7G, CT-EXO+agomiR-25-3p significantly exacerbated the impairment of osteogenic differentiation and autophagic activity in BMSCs, as evidenced by decreased expression of RUNX2 and LC3II: LC3I ratio, and increased expression of P62, compared to BMSCs treated solely with CT-EXO. Conversely, antagomiR-25-3p notably reversed the impairment of osteogenic differentiation and autophagic activity in BMSCs induced by CT-EXO (Fig. 7G). Alizarin Red staining further confirmed that miR-25-3p inhibited mineralized deposits (Fig. 7H–J). Thus, the results indicate that miR-25-3p plays a crucial role in the CT-EXO-induced inhibition of osteogenic differentiation and autophagic activity in BMSCs.

miRNAs regulate the expression of their target genes by interacting with the 3′-untranslated region (3′-UTR) or protein-coding sequence of target mRNAs. Using various bioinformatic target prediction techniques, we searched for targets of miR-25-3p that could be involved in the regulation of osteogenic differentiation and autophagic activity in BMSCs (Fig. 7K). SATB2 was suggested as a potential target of miR-25-3p. (Fig. 7L). To assess whether miR-25-3p can directly bind to the 3′-UTR of SATB2, luciferase reporter constructs were generated containing either wild-type (WT) or mutant (MUT) versions of the anticipated miRNA-binding sites within the SATB2 sequence (designated as WT-pGL3-SATB2 and MUT-pGL3-SATB2, respectively). Subsequently, the impact of miR-25-3p on luciferase enzyme activity in BMSCs was examined following transfection of WT-pGL3-SATB2 and MUT-pGL3-SATB2 with miR-25-3p mimics. The luciferase activity of the SATB2 3′-UTR reporter gene was suppressed by miR-25-3p mimics, whereas MUT-pGL3-SATB2 abrogated this effect entirely (Fig. 7L). These results suggest that miR-25-3p specifically targets the 3′-UTR of SATB2. Moreover, miR-25-3p levels in BMSCs were significantly increased by mimics or decreased by inhibitors, as depicted in Fig. 7M, which was confirmed by qRT-PCR. Additionally, we demonstrated that overexpression of miR-25-3p in BMSCs led to a reduction in SATB2 expression, while inhibition of miR-25-3p resulted in increased SATB2 expression (Fig. 7N). Furthermore, we observed that autophagic activity and osteogenic differentiation of BMSCs were suppressed by miR-25-3p overexpression but enhanced by miR-25-3p downregulation (Fig. S14A–D).

To investigate SATB2 function in miR-25-3p-controlled osteogenesis and autophagy, the expression of SATB2 was knocked down in BMSCs by transfection with SATB2 small interfering RNA (siSATB2). Western blotting analysis revealed that transfection of BMSCs with siSATB2 effectively reduced SATB2 protein expression (Fig. 7O). Since siSATB2#3 demonstrated the highest knockdown efficiency, it was chosen for the next experiment. Decreased expression of RUNX2 and mineralized deposit were further indicators that SATB2 knockdown reduced osteogenic differentiation (Fig. 7P–S). Autophagic activity was also reduced by knocking down SATB2, as measured by a greater amount of P62 and a lower LC3 II ：LC3 I ratio (Fig. 7P). Then we determined whether knocking down SATB2 could abolish the function of the miR-25-3p inhibitor. Fig. 7T reveals that miR-25-3p inhibitor significantly increased the expression of RUNX2 and LC3 II ：LC3 I ratio, markedly decreasing the expression of P62, the same as the previous results. However, the increase of BMSCs osteogenic differentiation and autophagic activity was reversed by siSATB2 treatment (Fig. 7T). Alizarin Red staining also confirmed that miR-25-3p inhibitor increased mineralized deposit, but knocking down SATB2 abolished this effect (Fig. 7U–W).

These findings indicate that miR-25-3p, enriched in CT-EXO, attenuate osteogenic differentiation in BMSCs by inhibiting their autophagic activity. Furthermore, our research identified SATB2 as the target gene for miR-25-3p, demonstrating that miR-25-3p can suppress the autophagic activity and osteogenic differentiation of BMSCs by downregulating SATB2 expression.

**Inhibition of miR-25-3p level** **alleviated CT-induced bone loss in mice**

To further investigate the impact of miR-25-3p on CT-induced bone loss, CT mice were treated with vehicle, antagomiR-NC, or antagomiR-25-3p once every 2 weeks for 8 weeks via intramedullary injection (Fig. 8A). AntagomiR-25-3p significantly mitigated CT-induced bone loss, resulting in increased BMD, Tb. BV/TV, and Tb. N, along with decreased Tb. Sp, compared to the antagomiR-NC-treated group (Fig. 8B–G). AntagomiR-25-3p also increased Ct. Th but not Ct. Ar/Tt. Ar, compared with the antagomiR-NC-treated group (Fig. 8H, I). In CT mice treated with antagomiR-25-3p, new bone formation and mineralization were enhanced, as observed through calcein double labeling, compared to the antagomiR-NC-treated group (Fig. 8J–L). Additionally, antagomiR-25-3p increased the number of osteoblasts on the trabecular bone surface of CT mice, as depicted in Fig. 8M and N, in comparison to the antagomiR-NC-treated group. Moreover, TRAP staining demonstrated that inhibition of miR-25-3p resulted in a decrease in the number of osteoclasts compared to the antagomiR-NC-treated group (Fig. 8O, P). AntagomiR-25-3p also increased the length of the femur in CT-induced mice, compared with the antagomiR-NC-treated group (Fig. S15A, B). Additionally, antagomiR-25-3p increased the body weight of CT mice compared with the antagomiR-NC-treated group (Fig. S15C). Furthermore, we administered the drug via tail vein injection in mice (Fig. S16A). Consistent with previous results, antagomiR-25-3p significantly increased bone mass in CT mice (Fig. S16B-I). In summary, all these findings indicate that miR-25-3p plays a crucial role in CT-induced bone loss.

**Discussion**

In our study, we discovered that cold exposure induces bone loss and impairs autophagic activity. Additionally, we found that plasma-derived exosomes mediate cold exposure-induced bone loss, with miR-25-3p, enriched in CT-EXO, playing a significant role in this process. Moreover, we elucidated that miR-25-3p may be transported by CT-EXO to suppress osteogenic differentiation and autophagy in BMSCs by targeting SATB2. Importantly, we demonstrated that exosomal miR-25-3p from cold exposure-induced plasma impairs bone metabolism both in vitro and in vivo. These findings offer new insights into the molecular mechanisms underlying bone loss induced by exposure to cold temperatures.

In recent years, it has become increasingly evident that environmental temperature profoundly impacts the body's metabolism. Prolonged exposure to cold temperatures has been shown to alter the metabolism of glucose, lipids, and even bone[[42-44](#_ENREF_42)]. By inhibiting cartilage formation, cold exposure may shorten the limbs of homeotherms[[10](#_ENREF_10)]. Studies done so far provide strong evidence that warmth reduces bone loss by influencing the gut microbiome [[9](#_ENREF_9)]. Du et al. found that brown adipose tissue rescues bone loss induced by cold exposure after cold exposure for 1 month [[45](#_ENREF_45)]. Our findings indicate that a 2-month cold exposure treatment significantly reduces bone mass. Furthermore, our data revealed that cold exposure also increases the senescence of bone tissues. Numerous studies have demonstrated that the senescence of bone and the onset of osteopenia are associated with decreased autophagic activity[[27](#_ENREF_27), [28](#_ENREF_28), [46](#_ENREF_46)]. From our results, we also established that cold exposure can inhibit bone autophagy. These findings offer novel evidence suggesting that cold exposure promotes bone aging by suppressing autophagic activity, thereby contributing to bone loss.

Exosomes have been regarded as key mediators of cell–cell communication since they carry numerous molecules such as proteins, lipids, and RNA[[18](#_ENREF_18)]. Exosomes can be detected in various bodily fluids, including plasma, saliva, breast milk, sweat, tears, and urine [[47-50](#_ENREF_47)]. Recent research found that plasma-derived exosomes are involved in bone metabolism [[51](#_ENREF_51)]. In our study, we identified exosomes based on several criteria including shape, size, and the presence of exosomal markers. Additionally, DiO-labelling of exosomes showed that these plasma-derived exosomes were integrated by BMSCs. We observed that CT-EXO decreased BMSCs' osteogenic differentiation and promoted senescence. Moreover, our findings suggested that CT-EXO may inhibit BMSCs' autophagy. However, treatment with RAP reversed the effects of CT-EXO on BMSCs, restoring their osteogenic differentiation and autophagy capacity, while also attenuating the aging process. Previous work has shown that extracellular vesicles can be delivered to bone tissues[[2](#_ENREF_2)]. Our present study revealed that CT-EXO can also be transported to bone tissues., where they contribute to the impairment of bone mass. Interestingly, we observed that inhibiting exosome release by GW4869 partially reversed CT-induced bone loss. Furthermore, treatment with RAP prevented the detrimental effects of CT-EXO on bone mass. Remarkably, RT-EXO promoted the osteogenic differentiation of BMSCs, increased bone mass in RT-exposed mice, and mitigated bone loss in CT-exposed mice. This effect may be attributed to the plasma source of the RT-EXO we extracted, which originated from 20-week-old mice and demonstrated the ability to enhance the osteogenic differentiation of BMSCs[[52](#_ENREF_52)]. Therefore, CT-EXO mediates cold exposure-induced bone damage by inhibiting the autophagy of BMSCs.

It was recently discovered that miR-25-3p has a significant role in osteogenic differentiation. However, the specific effect of miR-25-3p in osteogenic differentiation has caused great controversy. According to one study, miR-25-3p promotes BMSCs proliferation and osteogenic differentiation[[53](#_ENREF_53)]. Linc02349, on the other hand, is an endogenous RNA that competes with miR-25-3p to promote osteogenesis, according to another study [[54](#_ENREF_54)]. In this study, we confirmed the enrichment of miR-25-3p in CT-EXO, which significantly reduced the osteogenic differentiation of BMSCs. Furthermore, BMSCs were treated with CT-EXO transfected with either agomiR-25-3p or antagomiR-25-3p. Our data demonstrated that miR-25-3p decreased the osteoblastic differentiation capacity of BMSCs, as evidenced by decreased levels of RUNX2 and ARS. Additionally, we observed that miR-25-3p reduced the autophagic activity of BMSCs, as indicated by altered expression of P62 and LC3B. Subsequently, antagomiR-25-3p was injected into cold-exposed mice, revealing its ability to reverse cold exposure-induced bone loss. These data indicate that CT-induced exosomal miR-25-3p impairs osteogenic differentiation and autophagy both *in vitro* and *in vivo*.

Reportedly, SATB2 interacts directly with RUNX2 and ATF4, two transcription factors that influence osteoblast development, and increases their activity [[55](#_ENREF_55)]. Recent research suggests that SATB2 increases expression of genes involved in autophagy [[56](#_ENREF_56), [57](#_ENREF_57)]. In this study, we employed a luciferase reporter assay along with various bioinformatic target prediction methods to confirm SATB2 as a direct target of miR-25-3p. Western blot analysis revealed that increased miR-25-3p expression correlated with decreased SATB2 expression. In BMSCs transfected with an inhibitor of miR-25-3p, SATB2 expression was considerably lower than in control cells. SATB2 is involved in autophagy and osteogenic differentiation, although its molecular mechanism in both processes is unknown. As a result, we looked at how SATB2 knockdown by siRNA affected BMSCs. As anticipated, once SATB2 was knocked down in BMSCs, RUNX2 expression was dramatically downregulated, and BMSCs autophagic activity also reduced. Furthermore, the effects on the osteogenic differentiation and autophagic activity of miR-25-3p inhibitor-treated BMSCs were diminished by SATB2 knockdown, demonstrating that SATB2 mediates the impact of miR-25-3p on BMSCs’ osteogenic differentiation and autophagy.

**Conclusion**

Our present study suggests that cold exposure can induce bone loss (Fig. 8Q) These results show that CT-EXO-derived miR-25-3p inhibits osteogenic differentiation and autophagic activity both *in vivo* and *in vitro* (Fig. 8Q). Exosomal miR-25-3p and SATB2 are part of a complex regulatory network that, if better understood, might lead to the creation of effective therapies for preventing cold-induced bone loss.

**Declaration**

**Ethics approval and consent to participate**

All animal experiments were reviewed and approved by the Ethics Committee of the Second Xiang-Ya Hospital, Central South University. All the procedures conform to the NIH publication Guide for the Care and Use of Laboratory Animals (8th edition, 2011). All the animals were formally approved by the Ethics Committee of the Second Xiang-Ya Hospital, Central South University (No. 2022735).

**Consent for publication**

All authors agree for publication.

**Competing interests**

The authors declare that they have no conflict of interest.

**Fundings**

This study was supported by the National Natural Science Foundation of China (No. 82370892, 82070910, 82100944, 82200869 and 82100494), National Key Research & Development Program (No. 2021YFC2501701, 2023YFC2507600), National Clinical Key Specialties Major Research Projects (Z2023026). The Health Research Project in Hunan Province (No. 20231696, 202103062278), Health Research Project of Hunan Provincial Health Commission (W20243019), the Natural Science Foundation of Hunan Province (No. 2022JJ40721, 2022JJ40715), Kunming medicine joint special project（Project Number：202001AY070001-222）, and National Undergraduate Innovation Training Program of Central South University (Project Number: 2022105330213 and 2022105330235)

**Author contributions**

L-QY, Z-HL and L-ML conceived and designed the experiments. L-ML performed the experiments and prepared the figures. YW, Q-SX, M-HZ, and BG analyzed the

data. FX, XL, and F-X-L, S-KS provided technical support. L-QY and L-ML wrote the manuscript. W-LO-Y, Y-YW, K-XT, J-YD, Y-CC, and Z-AZ, S-YH, Y-LW, X-C, Z-JL, YP revised the manuscript. All authors read and approved the final manuscript.

**Acknowledgements**

We sincerely thank the animal care staff and technicians of the Animal Experimental Center of the Second Xiangya Hospital of Central South University for their care and special treatment of these mice.

**References**

1. Liu H, Song P, Zhang H, Zhou F, Ji N, Wang M, Zhou G, Han R, Liu X, Weng W, et al: **Synthetic biology-based bacterial extracellular vesicles displaying BMP-2 and CXCR4 to ameliorate osteoporosis.** *J Extracell Vesicles* 2024, **13:**e12429.

2. Liu JH, Chen CY, Liu ZZ, Luo ZW, Rao SS, Jin L, Wan TF, Yue T, Tan YJ, Yin H, et al: **Extracellular Vesicles from Child Gut Microbiota Enter into Bone to Preserve Bone Mass and Strength.** *Adv Sci (Weinh)* 2021, **8:**2004831.

3. Stearns SC: **Life history evolution: successes, limitations, and prospects.** *Naturwissenschaften* 2000, **87:**476-486.

4. Sun W, Dong H, Becker AS, Dapito DH, Modica S, Grandl G, Opitz L, Efthymiou V, Straub LG, Sarker G, et al: **Cold-induced epigenetic programming of the sperm enhances brown adipose tissue activity in the offspring.** *Nat Med* 2018, **24:**1372-1383.

5. Ziętak M, Kovatcheva-Datchary P, Markiewicz LH, Ståhlman M, Kozak LP, Bäckhed F: **Altered Microbiota Contributes to Reduced Diet-Induced Obesity upon Cold Exposure.** *Cell Metab* 2016, **23:**1216-1223.

6. Worthmann A, John C, Rühlemann MC, Baguhl M, Heinsen FA, Schaltenberg N, Heine M, Schlein C, Evangelakos I, Mineo C, et al: **Cold-induced conversion of cholesterol to bile acids in mice shapes the gut microbiome and promotes adaptive thermogenesis.** *Nat Med* 2017, **23:**839-849.

7. Chevalier C, Stojanović O, Colin DJ, Suarez-Zamorano N, Tarallo V, Veyrat-Durebex C, Rigo D, Fabbiano S, Stevanović A, Hagemann S, et al: **Gut Microbiota Orchestrates Energy Homeostasis during Cold.** *Cell* 2015, **163:**1360-1374.

8. Spiljar M, Steinbach K, Rigo D, Suárez-Zamorano N, Wagner I, Hadadi N, Vincenti I, Page N, Klimek B, Rochat MA, et al: **Cold exposure protects from neuroinflammation through immunologic reprogramming.** *Cell Metab* 2021, **33:**2231-2246.e2238.

9. Chevalier C, Kieser S, Çolakoğlu M, Hadadi N, Brun J, Rigo D, Suárez-Zamorano N, Spiljar M, Fabbiano S, Busse B, et al: **Warmth Prevents Bone Loss Through the Gut Microbiota.** *Cell Metab* 2020, **32:**575-590.e577.

10. Serrat MA, King D, Lovejoy CO: **Temperature regulates limb length in homeotherms by directly modulating cartilage growth.** *Proc Natl Acad Sci U S A* 2008, **105:**19348-19353.

11. Serrat MA, Schlierf TJ, Efaw ML, Shuler FD, Godby J, Stanko LM, Tamski HL: **Unilateral heat accelerates bone elongation and lengthens extremities of growing mice.** *J Orthop Res* 2015, **33:**692-698.

12. Kalluri R, LeBleu VS: **The biology, function, and biomedical applications of exosomes.** *Science* 2020, **367**.

13. Lei LM, Lin X, Xu F, Shan SK, Guo B, Li FX, Zheng MH, Wang Y, Xu QS, Yuan LQ: **Exosomes and Obesity-Related Insulin Resistance.** *Front Cell Dev Biol* 2021, **9:**651996.

14. Xie C, Ji N, Tang Z, Li J, Chen Q: **The role of extracellular vesicles from different origin in the microenvironment of head and neck cancers.** *Mol Cancer* 2019, **18:**83.

15. Behera J, Kumar A, Voor MJ, Tyagi N: **Exosomal lncRNA-H19 promotes osteogenesis and angiogenesis through mediating Angpt1/Tie2-NO signaling in CBS-heterozygous mice.** *Theranostics* 2021, **11:**7715-7734.

16. Qi X, Zhang J, Yuan H, Xu Z, Li Q, Niu X, Hu B, Wang Y, Li X: **Exosomes Secreted by Human-Induced Pluripotent Stem Cell-Derived Mesenchymal Stem Cells Repair Critical-Sized Bone Defects through Enhanced Angiogenesis and Osteogenesis in Osteoporotic Rats.** *Int J Biol Sci* 2016, **12:**836-849.

17. Zhang L, Jiao G, Ren S, Zhang X, Li C, Wu W, Wang H, Liu H, Zhou H, Chen Y: **Exosomes from bone marrow mesenchymal stem cells enhance fracture healing through the promotion of osteogenesis and angiogenesis in a rat model of nonunion.** *Stem Cell Res Ther* 2020, **11:**38.

18. Bagherniya M, Butler AE, Barreto GE, Sahebkar A: **The effect of fasting or calorie restriction on autophagy induction: A review of the literature.** *Ageing Res Rev* 2018, **47:**183-197.

19. White E, Lattime EC, Guo JY: **Autophagy Regulates Stress Responses, Metabolism, and Anticancer Immunity.** *Trends Cancer* 2021, **7:**778-789.

20. Deretic V: **Autophagy in inflammation, infection, and immunometabolism.** *Immunity* 2021, **54:**437-453.

21. Menzies FM, Fleming A, Caricasole A, Bento CF, Andrews SP, Ashkenazi A, Füllgrabe J, Jackson A, Jimenez Sanchez M, Karabiyik C, et al: **Autophagy and Neurodegeneration: Pathogenic Mechanisms and Therapeutic Opportunities.** *Neuron* 2017, **93:**1015-1034.

22. Fleming A, Bourdenx M, Fujimaki M, Karabiyik C, Krause GJ, Lopez A, Martín-Segura A, Puri C, Scrivo A, Skidmore J, et al: **The different autophagy degradation pathways and neurodegeneration.** *Neuron* 2022, **110:**935-966.

23. DeSelm CJ, Miller BC, Zou W, Beatty WL, van Meel E, Takahata Y, Klumperman J, Tooze SA, Teitelbaum SL, Virgin HW: **Autophagy proteins regulate the secretory component of osteoclastic bone resorption.** *Dev Cell* 2011, **21:**966-974.

24. Owen HC, Vanhees I, Gunst J, Van Cromphaut S, Van den Berghe G: **Critical illness-induced bone loss is related to deficient autophagy and histone hypomethylation.** *Intensive Care Med Exp* 2015, **3:**52.

25. Shapiro IM, Layfield R, Lotz M, Settembre C, Whitehouse C: **Boning up on autophagy: the role of autophagy in skeletal biology.** *Autophagy* 2014, **10:**7-19.

26. Cinque L, Forrester A, Bartolomeo R, Svelto M, Venditti R, Montefusco S, Polishchuk E, Nusco E, Rossi A, Medina DL, et al: **FGF signalling regulates bone growth through autophagy.** *Nature* 2015, **528:**272-275.

27. Hansen M, Rubinsztein DC, Walker DW: **Autophagy as a promoter of longevity: insights from model organisms.** *Nat Rev Mol Cell Biol* 2018, **19:**579-593.

28. Chen K, Yang YH, Jiang SD, Jiang LS: **Decreased activity of osteocyte autophagy with aging may contribute to the bone loss in senile population.** *Histochem Cell Biol* 2014, **142:**285-295.

29. Ma Y, Qi M, An Y, Zhang L, Yang R, Doro DH, Liu W, Jin Y: **Autophagy controls mesenchymal stem cell properties and senescence during bone aging.** *Aging Cell* 2018, **17**.

30. Luo D, Ren H, Li T, Lian K, Lin D: **Rapamycin reduces severity of senile osteoporosis by activating osteocyte autophagy.** *Osteoporos Int* 2016, **27:**1093-1101.

31. Onal M, Piemontese M, Xiong J, Wang Y, Han L, Ye S, Komatsu M, Selig M, Weinstein RS, Zhao H, et al: **Suppression of autophagy in osteocytes mimics skeletal aging.** *J Biol Chem* 2013, **288:**17432-17440.

32. Rupaimoole R, Slack FJ: **MicroRNA therapeutics: towards a new era for the management of cancer and other diseases.** *Nat Rev Drug Discov* 2017, **16:**203-222.

33. Brennan GP, Henshall DC: **MicroRNAs as regulators of brain function and targets for treatment of epilepsy.** *Nat Rev Neurol* 2020, **16:**506-519.

34. Ying W, Riopel M, Bandyopadhyay G, Dong Y, Birmingham A, Seo JB, Ofrecio JM, Wollam J, Hernandez-Carretero A, Fu W, et al: **Adipose Tissue Macrophage-Derived Exosomal miRNAs Can Modulate In Vivo and In Vitro Insulin Sensitivity.** *Cell* 2017, **171:**372-384.e312.

35. Qin W, Dallas SL: **Exosomes and Extracellular RNA in Muscle and Bone Aging and Crosstalk.** *Curr Osteoporos Rep* 2019, **17:**548-559.

36. Xie Y, Chen Y, Zhang L, Ge W, Tang P: **The roles of bone-derived exosomes and exosomal microRNAs in regulating bone remodelling.** *J Cell Mol Med* 2017, **21:**1033-1041.

37. Thibonnier M, Ghosh S, Blanchard A: **Effects of a short-term cold exposure on circulating microRNAs and metabolic parameters in healthy adult subjects.** *J Cell Mol Med* 2022, **26:**548-562.

38. Zhu H, Guo ZK, Jiang XX, Li H, Wang XY, Yao HY, Zhang Y, Mao N: **A protocol for isolation and culture of mesenchymal stem cells from mouse compact bone.** *Nat Protoc* 2010, **5:**550-560.

39. Lin X, Zhan JK, Zhong JY, Wang YJ, Wang Y, Li S, He JY, Tan P, Chen YY, Liu XB, et al: **lncRNA-ES3/miR-34c-5p/BMF axis is involved in regulating high-glucose-induced calcification/senescence of VSMCs.** *Aging (Albany NY)* 2019, **11:**523-535.

40. Xie H, Cui Z, Wang L, Xia Z, Hu Y, Xian L, Li C, Xie L, Crane J, Wan M, et al: **PDGF-BB secreted by preosteoclasts induces angiogenesis during coupling with osteogenesis.** *Nat Med* 2014, **20:**1270-1278.

41. Tkach M, Théry C: **Communication by Extracellular Vesicles: Where We Are and Where We Need to Go.** *Cell* 2016, **164:**1226-1232.

42. Seki T, Yang Y, Sun X, Lim S, Xie S, Guo Z, Xiong W, Kuroda M, Sakaue H, Hosaka K, et al: **Brown-fat-mediated tumour suppression by cold-altered global metabolism.** *Nature* 2022, **608:**421-428.

43. Okamatsu-Ogura Y, Kuroda M, Tsutsumi R, Tsubota A, Saito M, Kimura K, Sakaue H: **UCP1-dependent and UCP1-independent metabolic changes induced by acute cold exposure in brown adipose tissue of mice.** *Metabolism* 2020, **113:**154396.

44. Wee NKY, Nguyen AD, Enriquez RF, Zhang L, Herzog H, Baldock PA: **Neuropeptide Y Regulation of Energy Partitioning and Bone Mass During Cold Exposure.** *Calcif Tissue Int* 2020, **107:**510-523.

45. Du J, He Z, Xu M, Qu X, Cui J, Zhang S, Zhang S, Li H, Yu Z: **Brown Adipose Tissue Rescues Bone Loss Induced by Cold Exposure.** *Front Endocrinol (Lausanne)* 2021, **12:**778019.

46. Sacitharan PK: **Ageing and Osteoarthritis.** *Subcell Biochem* 2019, **91:**123-159.

47. Lässer C, Alikhani VS, Ekström K, Eldh M, Paredes PT, Bossios A, Sjöstrand M, Gabrielsson S, Lötvall J, Valadi H: **Human saliva, plasma and breast milk exosomes contain RNA: uptake by macrophages.** *J Transl Med* 2011, **9:**9.

48. Mleczko J, Ortega FJ, Falcon-Perez JM, Wabitsch M, Fernandez-Real JM, Mora S: **Extracellular Vesicles from Hypoxic Adipocytes and Obese Subjects Reduce Insulin-Stimulated Glucose Uptake.** *Mol Nutr Food Res* 2018, **62**.

49. Wu CX, Liu ZF: **Proteomic Profiling of Sweat Exosome Suggests its Involvement in Skin Immunity.** *J Invest Dermatol* 2018, **138:**89-97.

50. Inubushi S, Kawaguchi H, Mizumoto S, Kunihisa T, Baba M, Kitayama Y, Takeuchi T, Hoffman RM, Tanino H, Sasaki R: **Oncogenic miRNAs Identified in Tear Exosomes From Metastatic Breast Cancer Patients.** *Anticancer Res* 2020, **40:**3091-3096.

51. Tao SC, Yuan T, Rui BY, Zhu ZZ, Guo SC, Zhang CQ: **Exosomes derived from human platelet-rich plasma prevent apoptosis induced by glucocorticoid-associated endoplasmic reticulum stress in rat osteonecrosis of the femoral head via the Akt/Bad/Bcl-2 signal pathway.** *Theranostics* 2017, **7:**733-750.

52. Xun J, Li C, Liu M, Mei Y, Zhou Q, Wu B, Xie F, Liu Y, Dai R: **Serum exosomes from young rats improve the reduced osteogenic differentiation of BMSCs in aged rats with osteoporosis after fatigue loading in vivo.** *Stem Cell Res Ther* 2021, **12:**424.

53. Feng X, Xiang Q, Jia J, Guo T, Liao Z, Yang S, Cai X, Liu X: **CircHGF suppressed cell proliferation and osteogenic differentiation of BMSCs in ONFH via inhibiting miR-25-3p binding to SMAD7.** *Mol Ther Nucleic Acids* 2022, **28:**99-113.

54. Cao L, Liu W, Zhong Y, Zhang Y, Gao D, He T, Liu Y, Zou Z, Mo Y, Peng S, Shuai C: **Linc02349 promotes osteogenesis of human umbilical cord-derived stem cells by acting as a competing endogenous RNA for miR-25-3p and miR-33b-5p.** *Cell Prolif* 2020, **53:**e12814.

55. Dobreva G, Chahrour M, Dautzenberg M, Chirivella L, Kanzler B, Fariñas I, Karsenty G, Grosschedl R: **SATB2 is a multifunctional determinant of craniofacial patterning and osteoblast differentiation.** *Cell* 2006, **125:**971-986.

56. Dong W, Zhang P, Fu Y, Ge J, Cheng J, Yuan H, Jiang H: **Roles of SATB2 in site-specific stemness, autophagy and senescence of bone marrow mesenchymal stem cells.** *J Cell Physiol* 2015, **230:**680-690.

57. Zhang S, He W, Li A, Zhao C, Chen Y, Xu C, Zhang Q, Zheng D, Chen M, Miao H, Huang Y: **Involvement of the TNF-α/SATB2 axis in the induced apoptosis and inhibited autophagy of osteoblasts by the antipsychotic Risperidone.** *Mol Med* 2022, **28:**46.

**Fig. 1 Cold exposure induced bone loss and altered bone metabolism in mice.**

**A**, The schematic flow diagram represents the *in vivo* treatment of CT or RT. n = 5 per group. **B**, Representative micro-CT images of trabecular (top) and cortical (bottom) bone in RT- or CT-treated mice. Scale bars represent 500 µm (top) and 1 mm (bottom). **C–I**, Parameters of bone mass analysed by μCT: BMD, bone mineral density; Tb. BV/TV, bone volume over tissue volume; Tb. Th, trabecular thickness; Tb. N, trabecular number; Tb. Sp, trabecular separation; Ct. Ar/Tt. Ar, cortical bone area fraction; Ct. Th, cortical thickness. n = 5 per group. **J**, Calcein double labelling images of the mineralized surface of mouse femora. Scale bar represents 50 µm. **K, L**, Parameters of bone formation. MAR, BFR/BS, n = 5 per group. **M**, Representative OCN-stained section with quantification of the (**N**) number of osteoblasts (N. OBs) on the trabecular bone surface (BS) in distal femora from mice treated with RT or CT. n = 5 per group. Scale bar represents 100 µm. **O**, Representative TRAP-stained sections with quantification of the (**P**) number of osteoclasts (N. OCs) on the trabecular bone surface (BS) in distal femora from mice treated with RT or CT. n = 5 per group. Scale bar represents 100 µm. * P < 0.05, ** P < 0.01, *** P < 0.001, **** P < 0.0001

**Fig. 2 Inhibition of exosomes release alleviated CT-induced bone loss.** **A**, Experimental design of the mice treated with CT+vehicle or CT+GW4869 by intraperitoneal injection (n = 5 per group). **B**, Representative µCT images of trabecular (top) and cortical (bottom) bone in CT+vehicle- or CT+GW4869-treated mice. Scale bars represent 500 µm (top) and 1 mm (bottom). **C–I**, Parameters of trabecular bone mass analysed by micro-CT: BMD, Tb. BV/TV, Tb. Th, Tb. N, Tb. Sp, Ct. Ar/Tt. Ar, Ct. Th. n = 5 per group. **J**, Calcein double labelling images of the mineralized surface of mouse femora. Scale bar represents 50 µm. **K, L**, Parameters of bone formation. MAR, mineral apposition rate; BFR/BS, bone formation rate//bone surface. n = 5 per group. **M**, Representative OCN-stained section. Scale bar represents 100 µm. **N**, Quantification of the number of osteoblasts (N. OBs) on the trabecular bone surface (BS) in distal femora. n = 5 per group. **O**, TRAP-stained sections. Scale bar represents 100 µm. **P**, quantification of the number of osteoclasts (N. OCs) on the trabecular bone surface (BS) in distal femora. n = 5 per group. The vehicle referred to is dimethyl sulfoxide (DMSO). * P < 0.05, ** P < 0.01, *** P < 0.001, **** P < 0.0001

**Fig. 3 CT-induced plasma can decrease bone mass, removal of exosomes can ameliorate CT-induced plasma-triggered osteopenia.** **A**, Schematic flow diagram representing mice treated with PBS, CT-plasma, or CT-plasma-EXO. n = 5 per group. **B**, Representative micro-CT images of trabecular (top) and cortical (bottom) bone in CT-plasma- or CT-plasma-EXO-treated mice. Scale bars represent 500 µm (top) and 1 mm (bottom). **C–I**, Parameters of trabecular bone mass analysed by micro-CT: BMD, Tb. BV/TV, Tb. Th, Tb. N, Tb. Sp, Ct. Ar/Tt. Ar, Ct. Th. n = 5 per group. **J**, Calcein double labelling images of the mineralized surface of mouse femora. Scale bar represents 50 µm. **K, L**, Parameters of bone formation. MAR, mineral apposition rate; BFR/BS, bone formation rate/bone surface. n = 5 per group. **M**, Representative OCN-stained section. Scale bar represents 100 µm. **N**, Quantification of the number of osteoblasts (N. OBs) on the trabecular bone surface (BS) in distal femora. n = 5 per group. **O**, TRAP-stained sections. Scale bar represents 100 µm. **P**, quantification of the number of osteoclasts (N. OCs) on the trabecular bone surface (BS) in distal femora. n = 5 per group. The control group were injected PBS. * P < 0.05, ** P < 0.01, *** P < 0.001, **** P < 0.0001

**Fig. 4 CT-EXO can be taken up by BMSCs and impair the osteogenic differentiation of BMSCs. A**, Representative image of the ultrastructure of exosomes observed by transmission electron microscopy. Scale bar represents 100 µm. **B**, Average particle size distribution of exosomes. **C**, Exosomes markers CD9, CD81, and TSG101 determined by western blotting. **D**, Representative fluorescence micrograph of DiO-labelled exosomes (green) internalized by primary BMSCs, while blue represents the nucleus. The labelled exosomes were co-incubated with BMSCs for 12 h. Scale bar represents 20 µm. **E**, Representative western blot image showing the effect of CT-EXO on the protein levels of RUNX2 in BMSCs after 48 h co-incubation. **F, G**, Representative image of microscopic view (F) and entire plate view (G)ARS staining of BMSCs after exosomes treatment. Scale bars represents 250 µm. **H**, Quantification of the ARS. n = 3 per group. **I**, Representative image of colony formation assay after BMSCs were treated with exosomes. **J**, Representative image of SA-β-gal staining of BMSCs after exosomes treatment. Scale bar represents 100 µm. OIM represent the osteogenesis induced medium, and the Control represent the BMSCs without using the osteogenesis induced medium. * P < 0.05, ** P < 0.01, *** P < 0.001, **** P < 0.0001

**Fig. 5 CT-EXO decreased bone mass and altered bone metabolism in mice.** **A**, Representative fluorescence image of exosomes distribution in mice 24 h after exosome injection. **B**, Representative *ex vivo* fluorescence image of exosomes distribution in femur 24 h after exosomes injection. **C**, Representative fluorescence micrograph showing the DiO-labelled exosomes (green fluorescence) in the femur section. Scale bar represents 100 µm. CB: cortical bone; TB: trabecular bone; BM: bone marrow. **D**, Schematic flow diagram representing mice treated with PBS, RT-EXO, and CT-EXO. n = 6 per group. **E**, Representative µCT images of trabecular (top) and cortical (bottom) bone in femora from mice in PBS, RT-EXO, and CT-EXO groups. Scale bars represent 500 µm (top) and 1 mm (bottom). **F–L**, Quantitative analysis of BMD; Tb. BV/TV, Tb. Th, Tb. N, Tb. Sp, Ct. Ar/Tt. Ar, Ct. Th. n = 6 per group. **M**, Calcein double labelling images of the mineralized surface of mouse femora. Scale bar represents 50 µm. **N, O**, Quantitation of BFR/BS and MAR. n = 6 per group. **P**, Representative OCN-stained sections with quantification of (**Q**) osteoblast number. Scale bar: 100 µm. n = 6 per group. **R**, Representative TRAP-stained sections with quantification of (**S**) osteoclast number. Scale bar represents 100 µm. n = 6 per group. * P < 0.05, ** P < 0.01, *** P < 0.001, **** P < 0.0001

**Fig. 6 CT-EXO impairs bone mass by inhibiting autophagy.** **A**, Representative western blot image showing the effect of cold exposure on the protein levels of P21, P62, and LC3 in bone tissues.**B**, Representative western blot image showing the expression protein levels of RUNX2, P62, and LC3，P21 from control-, Vehicle-, CT-EXO-, RAP-, and RAP+CT-EXO-treated BMSCs. **C, D**, Representative image of microscopic view (D) and entire plate view (E) ARS staining. Scale bar represents 250 µm. **E**, Quantification of ARS. n = 3 per group. **F**, Representative Electron Microscopy Images of control-, Vehicle-, CT-EXO-, RAP-, and RAP+CT-EXO-treated BMSCs. Scale bars represent 5 µm **G**, Representative image of SA-β-gal staining of BMSCs after exosomes treatment. Scale bar represents 100 µm. The control group represents the group without osteogenic induction, while the vehicle group represents the group undergoing osteogenic induction with solvent intervention. **H**, Schematic flow diagram representing mice treated with vehicle, CT-EXO, RAP, and RAP+CT-EXO. n = 6 per group. **I**, Representative µCT images of trabecular (top) and cortical (bottom) bone in femora from mice in the vehicle, CT-EXO, RAP, and RAP+CT-EXO groups. Scale bars represent 500 µm (top) and 1 mm (bottom). **J–P**, Quantitative analysis of BMD, Tb. BV/TV, Tb. Th, Tb. N, Tb. Sp, Ct. Ar/Tt. Ar, Ct. Th. n = 5 per group. **Q**, Representative calcein double labelling images of the mineralized surface of mouse femora. Scale bar represents 50 µm. **R, S**, Quantitation of MAR and BFR/BS. n = 5 per group. **T**, Representative OCN-stained section. Scale bar represents 100 µm. **U**, Quantification of the number of osteoblasts (N. OBs) on the trabecular bone surface (BS) in distal femora. n = 5 per group. **V**, TRAP-stained sections. Scale bar represents 100 µm. **W**, quantification of the number of osteoclasts (N. OCs) on the trabecular bone surface (BS) in distal femora. n = 5 per group. The vehicle referred to is DMSO. * P < 0.05, ** P < 0.01, *** P < 0.001, **** P < 0.0001

**Fig. 7 Exosomal miR-25-3p enriched in CT-EXO regulated osteogenic differentiation and autophagy by tageting SATB2 in BMSCs.** **A**, Volcano diagram of differential expression between between RT-EXO and CT-EXO miRNA according to microarray analysis. **B–F**, qRT-PCR quantitative results of miRNA (miR-205-5p, miR-30d-5p, miR-320-3p, miR-222-3p miR-25-3p) level of RT-EXO or CT-EXO. n = 3 per group. **G**, Representative western blot image showing the expression protein levels of RUNX2, P62, and LC3 from miR-25-3p knocked-in or miR-25-3p knocked-down CT-EXO-treated BMSCs. **H, I**, Representative image of microscopic view (H) and entire plate view (I) ARS staining from miR-25-3p knocked-in or miR-25-3p knocked-down CT-EXO-treated BMSCs. Scale bar represents 250 µm **J**, Quantification of ARS. n = 3 per group. **K**, Venn diagram of the predicted target gene of miR-25-3p from Target scan (blue), miRDB (red), autophagy(yellow), and osteogenesis (orange). **L**, Luciferase reporter assays were conducted using luciferase constructs carrying a WT or mutant SATB2 3′-UTR co-transfected into BMSCs with miR-25-3p mimics. Firefly luciferase activity was normalized to *Renilla* luciferase activity. n = 5 per group. **M**, Transfection efficiency of miR-25-3p detected by qRT-PCR. **N**, Representative western blot image showing the protein expression levels of SATB2 from miR-25-3p knocked-in miR-25-3p knocked-down BMSCs. **O**, Knockout efficiency of SATB2 in BMSCs by western blot. **P**, SATB2, Runx2, P62, and LC3 expression were measured in the BMSCs treated with siSATB2#3 or siRNA control. **Q, R**, Representative image of microscopic view (Q) and entire plate view (R)ARS staining. Scale bar represents 250 µm. **S**, Quantification of ARS. **T**, Representative western blot image showing the expression protein levels of SATB2, RUNX2, P62, and LC3 from the inhibitor-NC, inhibitor, inhibitor+siRNA-NC, inhibitor+siSATB2 groups. **U, V**, Representative image of microscopic view (U) and entire plate view (V)ARS staining. Scale bar represents 250 µm. **W**, Quantification of ARS. n = 3 per group. All groups' cells have received osteogenic induction, the control group represents the group undergoing osteogenic induction without any intervention. CT-EXO+AgomiR-NC and CT-EXO-AntagomiR-NC served as negative controls for CT-EXO+AgomiR-25-3p and CT-EXO-AntagomiR-25-3p. * P < 0.05, ** P < 0.01, *** P < 0.001, **** P < 0.0001

**Fig. 8 Inhibition of miR-25-3p level alleviated CT-induced bone loss.** **A**, Schematic flow diagram representing mice treated with CT+vehicle, CT+antagomiR-NC, and CT+antagomiR-25-3p. n = 5 per group. **B**, Representative µCT images of trabecular (top) and cortical (bottom) bone in CT+vehicle-, CT+antagomiR-NC-, and CT+antagomiR-25-3p-treated mice. Scale bars represent 500 µm (top) and 1 mm (bottom). **C–I**, Parameters of trabecular bone mass analysed by micro-CT: BMD, Tb. BV/TV, Tb. Th, Tb. N, Tb. Sp, Ct. Ar/Tt. Ar, Ct. Th. n = 5 per group. **J**, Calcein double labelling images of the mineralized surface of mouse femora. Scale bar represents 50 µm. **K, L**, Parameters of bone formation MAR, BFR/BS. n = 5 per group. **M**, Representative OCN-stained section. Scale bar represents 100 µm. **N**, Quantification of the number of osteoblasts (N. OBs) on the trabecular bone surface (BS) in distal femora. n = 5 per group. **O**, TRAP-stained sections. Scale bar represents 100 µm. **P**, quantification of the number of osteoclasts (N. OCs) on the trabecular bone surface (BS) in distal femora. n = 5 per group. **Q**, Mechanism of cold exposure induced bone loss. The vehicle referred to is PBS. * P < 0.05, ** P < 0.01, *** P < 0.001, **** P < 0.0001

Fig.1


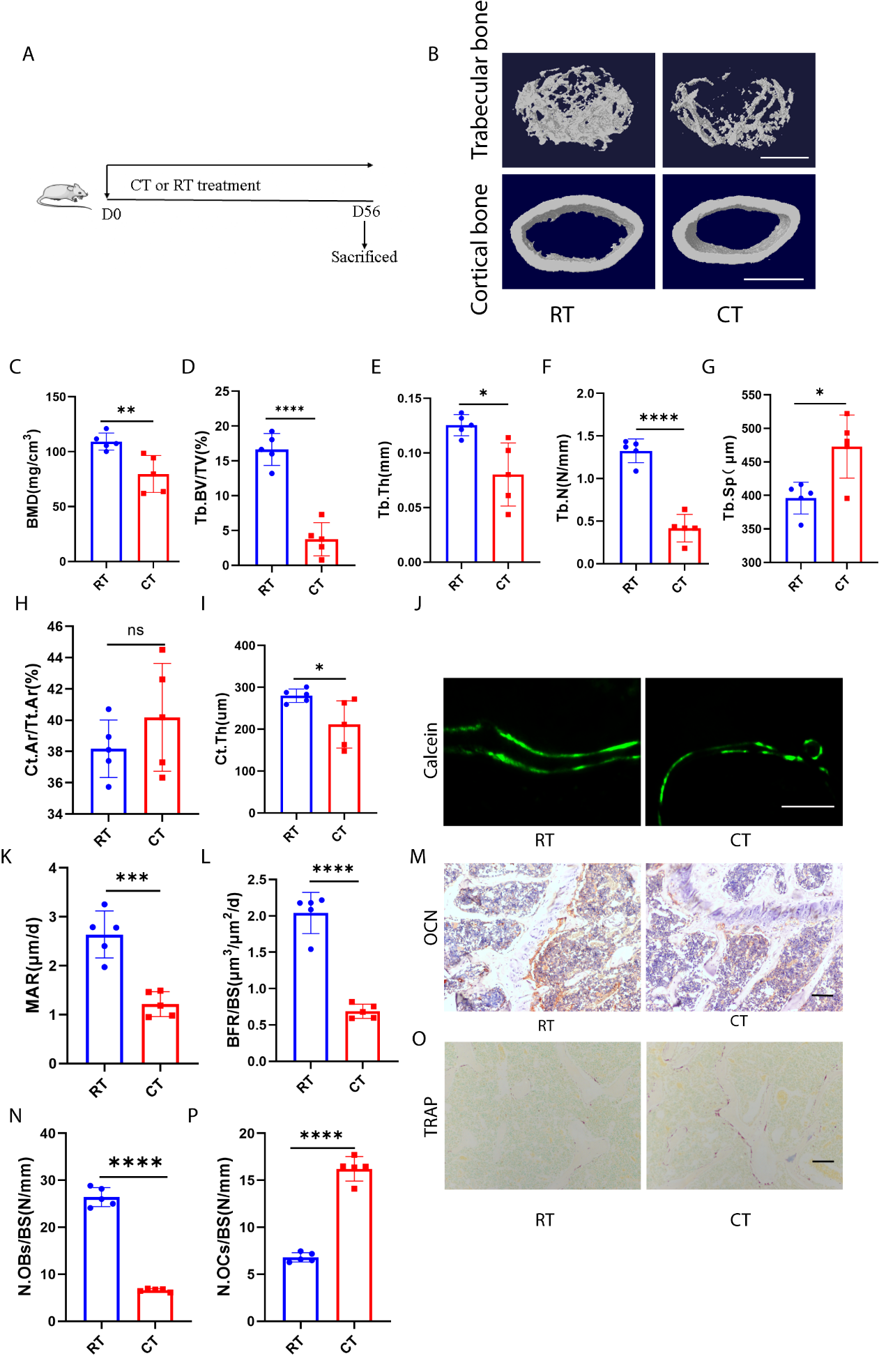


Fig.2


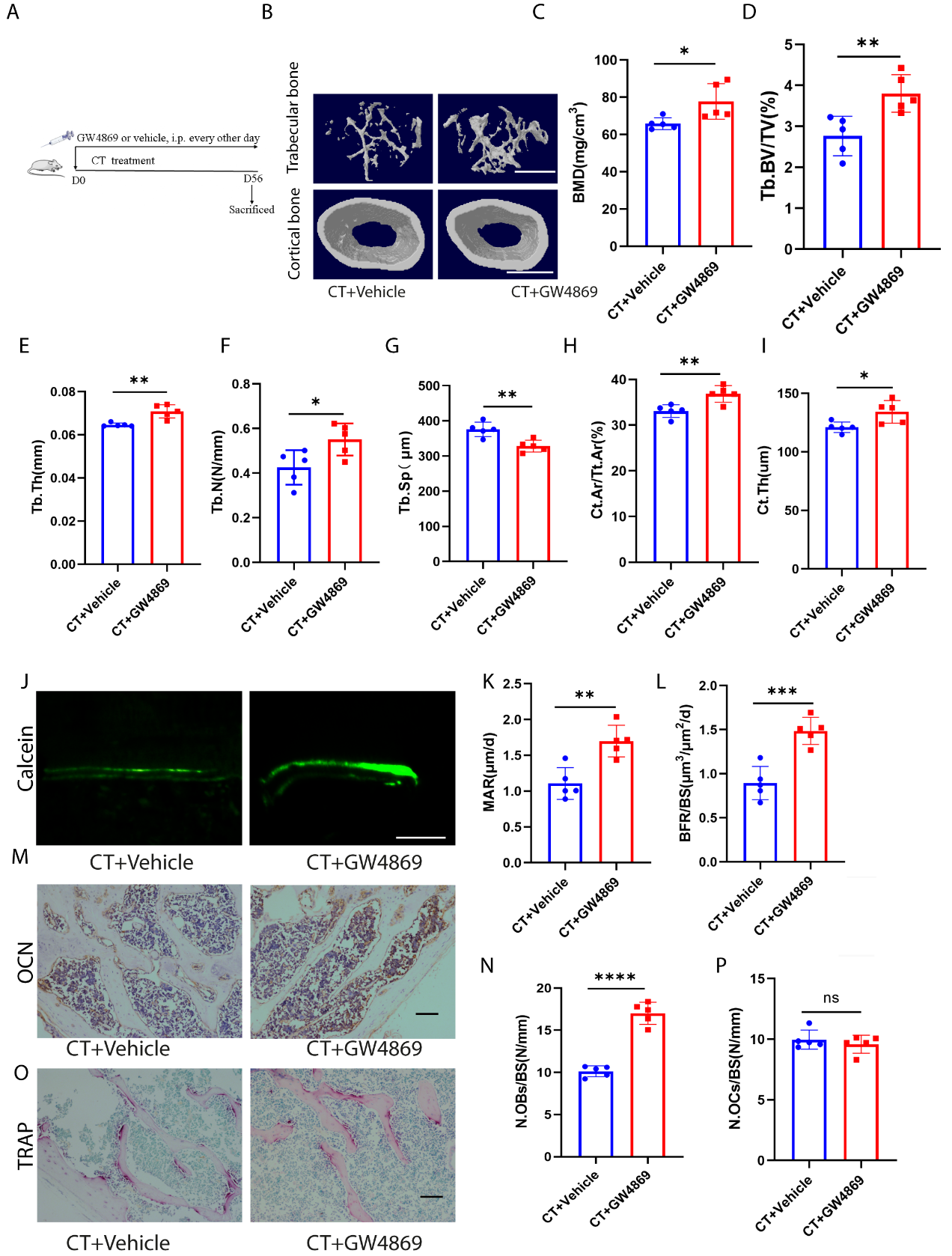


Fig.3


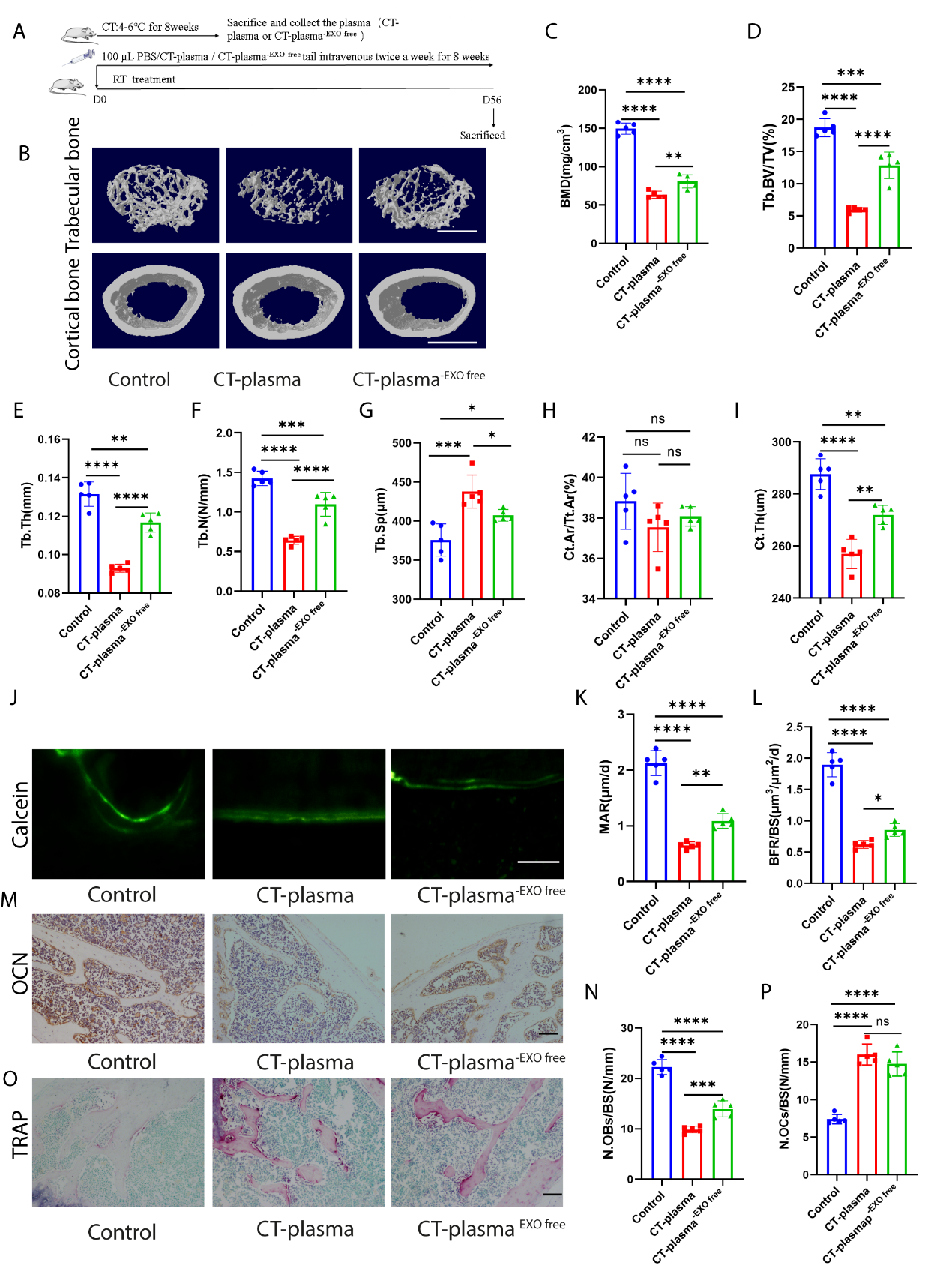


**Fig.4**


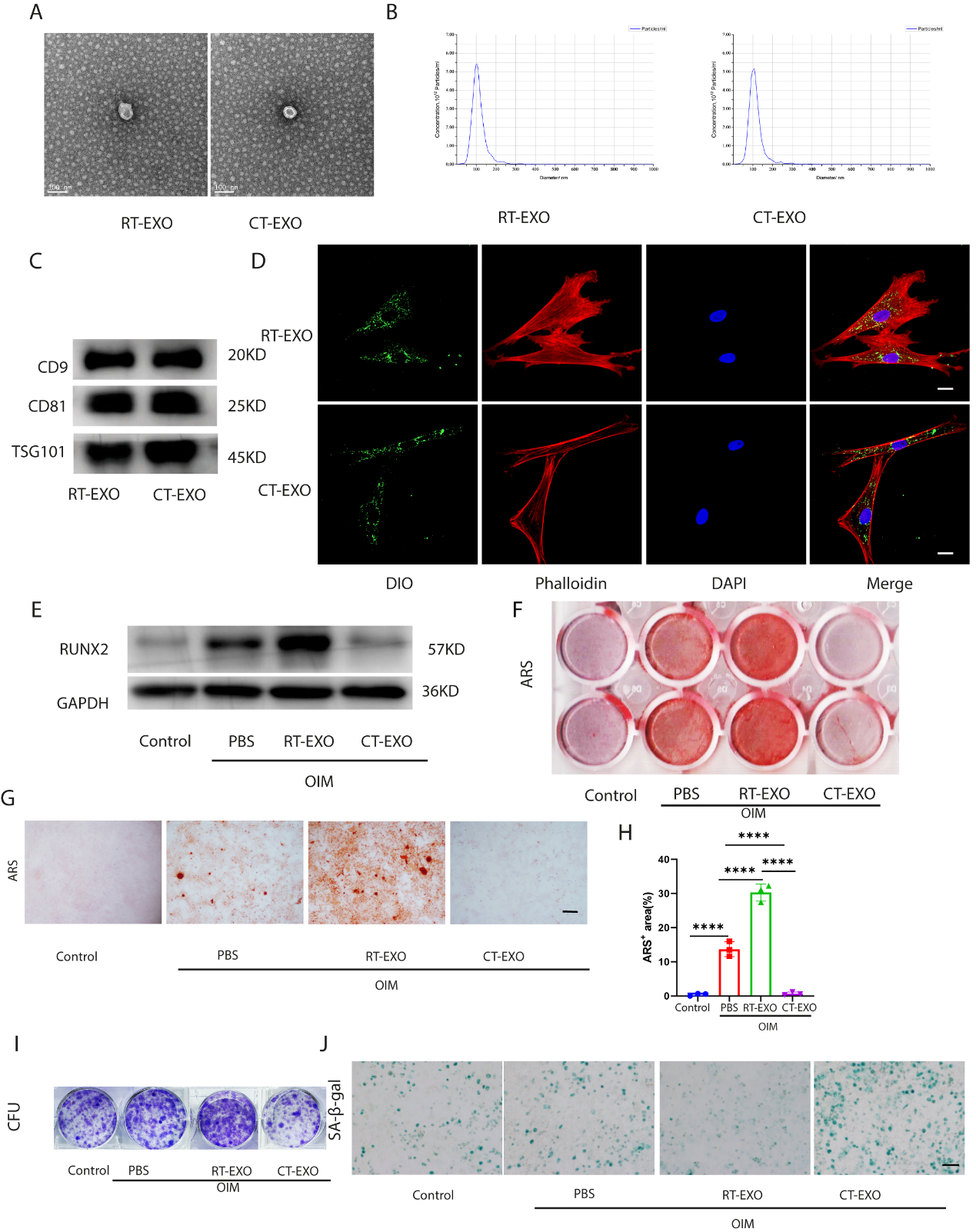


Fig.5


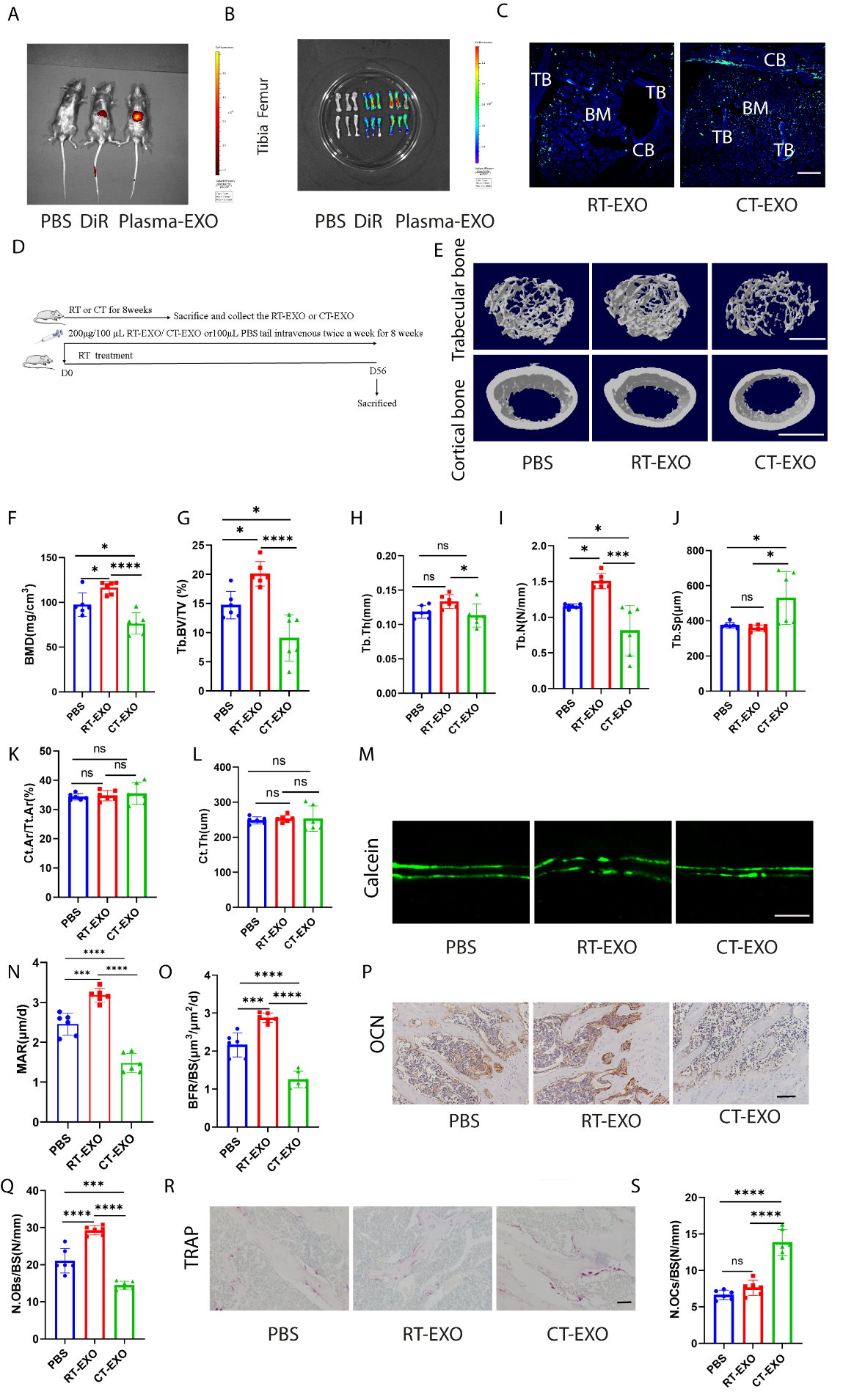


Fig.6


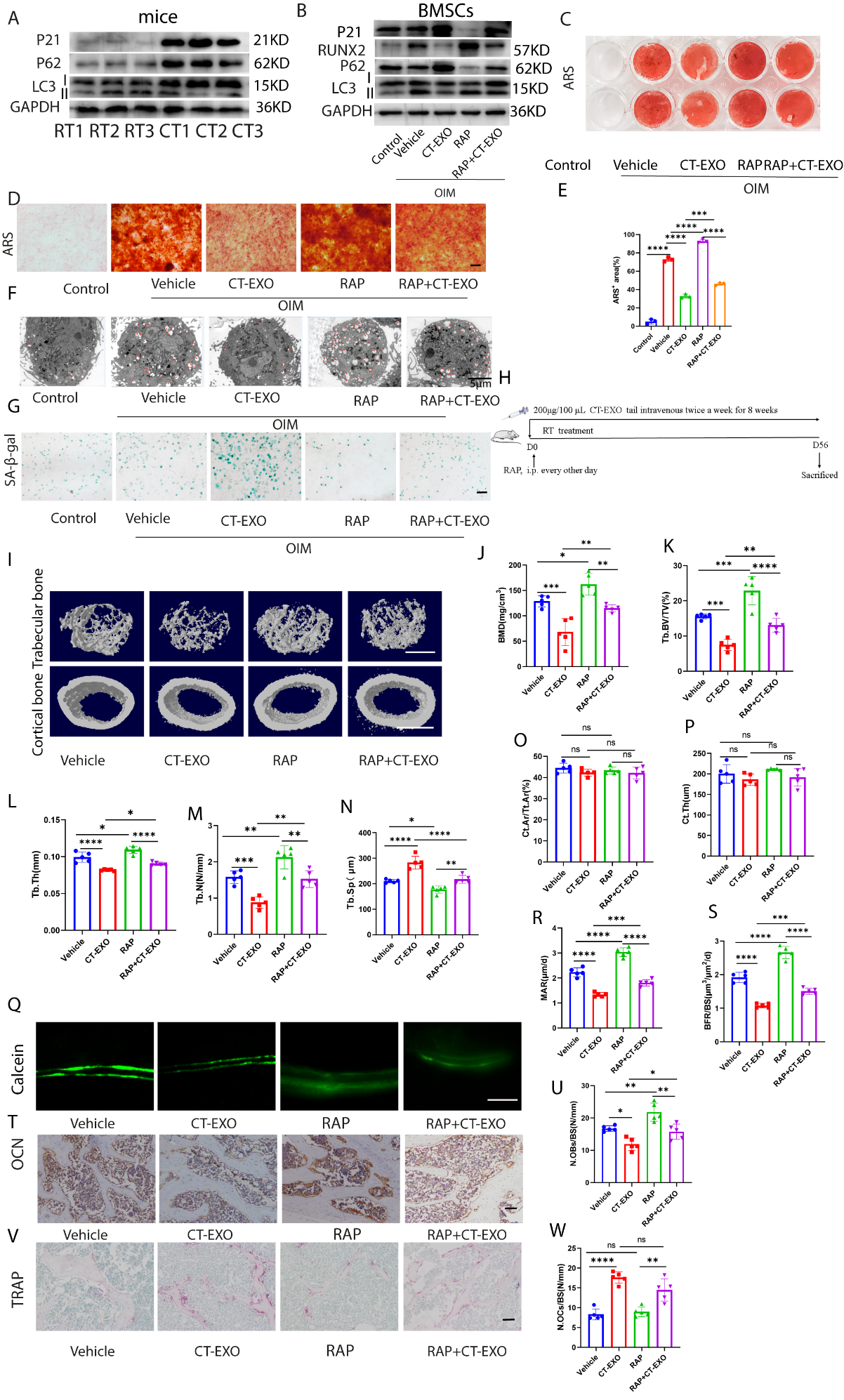


Fig.7


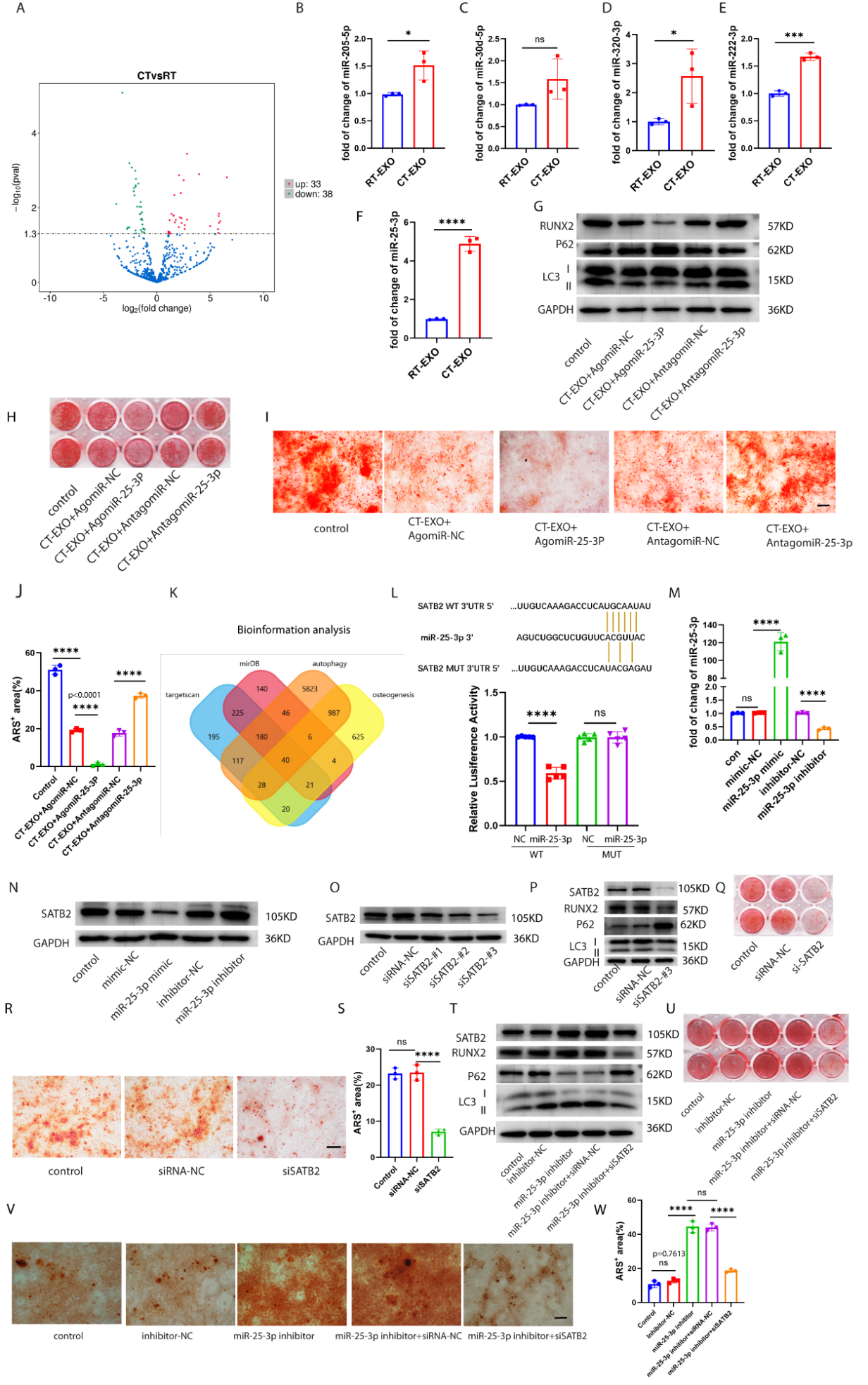


Fig.8


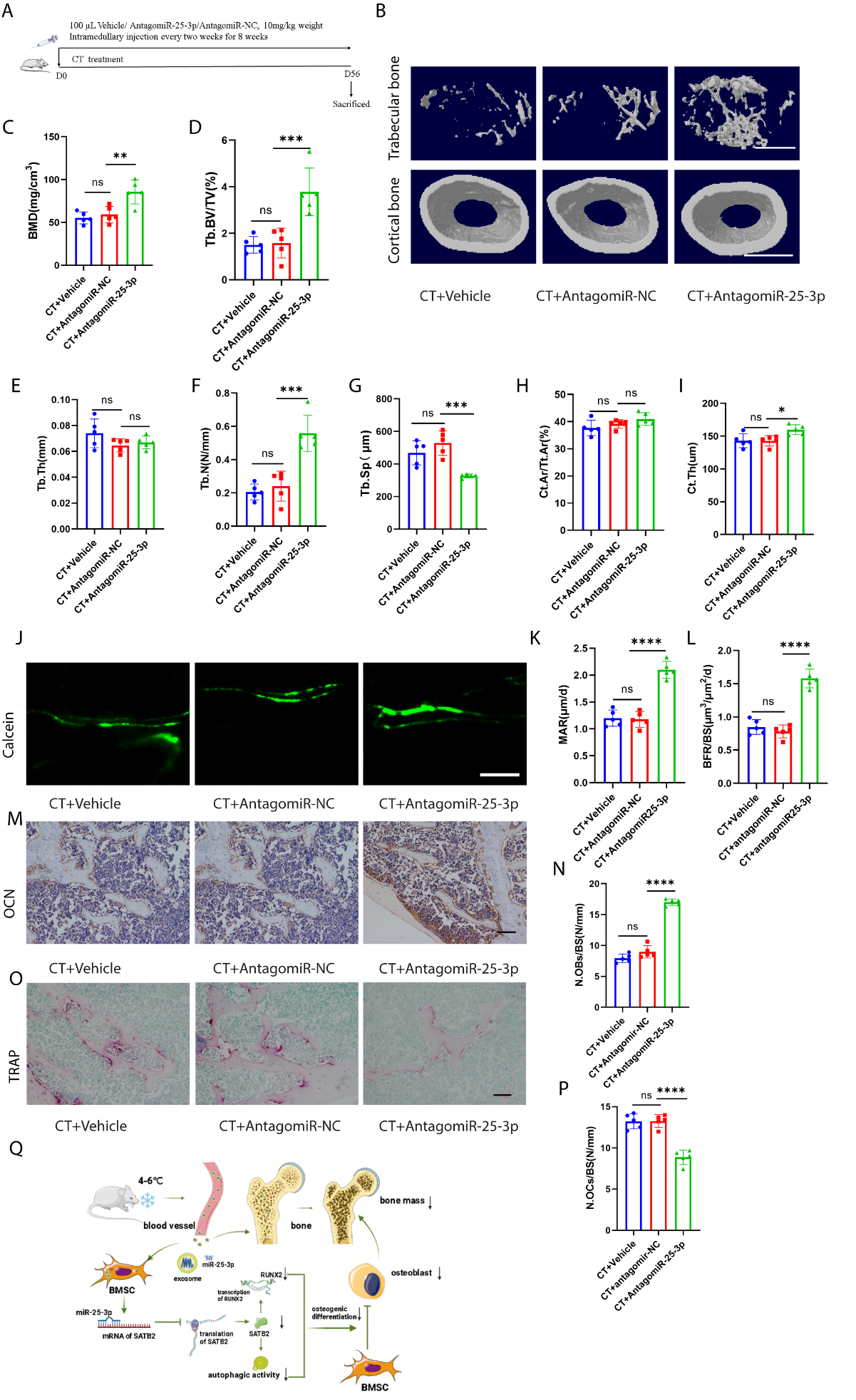

Supplement: Supplementary file 1 — Supplementary Material 1 [file 12951_2024_2640_MOESM1_ESM.docx]
